# Environmental Sensing and Borrower Feature Interaction for Uncertainty-Aware Loan Risk Prediction Under Extreme Climatic Conditions

**DOI:** 10.3390/s26206488

**Published:** 2026-10-13

**Authors:** Xinhan Wu, Jingyi Yuan, Lanteng Zhang, Biao Wu, Yongshun Zhang, Layun Yuan, Manzhou Li

**Affiliations:** 1 China Agricultural University, Beijing 100083, China; 2 Peking University, Beijing 100871, China; 3 Desert Comprehensive Management Center of Bayannur City, Bayannur 015099, China

**Keywords:** artificial intelligence-driven sensing, agricultural environmental sensing, multisource sensor data fusion, robust default prediction, predictive probability calibration

## Abstract

Extreme climatic events, including severe heatwaves, prolonged droughts, and anomalous flooding, can substantially disrupt agricultural production stability and further increase the risk of agricultural loan default. To address the limitations of conventional credit assessment in fully exploiting environmental sensing information, the scarcity of extreme-disaster samples, and fluctuations in sensing quality, a robust default risk prediction framework integrating agricultural environmental sensing observations, borrower-feature interactions, and uncertainty-aware learning is proposed. First, environmental exposure features and borrower-specific attributes are separately extracted through a dual-branch encoding network, and bidirectional cross-attention is employed to capture nonlinear interactions between climatic conditions and borrower risk. Subsequently, a climate-constrained disaster scenario augmentation mechanism is introduced to expand extreme-environment samples under regional, seasonal, crop-growth-stage, and inter-variable relationship constraints. A stabilized weighting strategy is further constructed by combining stochastic prediction with uncertainty estimation, thereby reducing the influence of highly noisy samples and anomalous observations on model training. Experimental results demonstrate that the proposed model achieves a PR-AUC of 0.791, a ROC-AUC of 0.909, and an F1-score of 0.781 under the combined evaluation of normal and extreme climatic conditions, while attaining a Brier Score of 0.098 and an ECE of 0.027. Under degraded sensing conditions involving missingness, noise, and bias, the model maintains high discriminative capability (PR-AUC of 0.758, ROC-AUC of 0.891, and F1-score of 0.756) and well-calibrated risk estimates (Brier Score of 0.112 and ECE of 0.031). Disaggregated evaluations across perturbation types, sensitivity analyses regarding spatial matching and synthetic labeling, external temporal holdout and cross-regional validations, and post-hoc feature attribution analyses further confirm the robustness and practical interpretability of the framework. Overall, the proposed framework consistently outperforms Logistic Regression, SVM, Random Forest, XGBoost, LightGBM, DNN, LSTM, Deep Ensemble, and the previous model.

## 1. Introduction

Agricultural production is highly dependent on environmental conditions, and climatic variations directly affect production processes, crop yields, and income stability [1]. Global climate change has increased the frequency and intensity of severe heatwaves, prolonged droughts, and anomalous flooding, exposing agricultural operators to greater uncertainty and risk [2]. In agricultural lending, such events can cause production losses and impair operating income, cash-flow stability, and loan repayment capacity [3]. Therefore, credit assessment based primarily on borrower information may fail to capture climate-driven changes in agricultural risk, making it necessary to jointly consider borrower characteristics and environmental exposure [4].

Advances in agricultural Internet of Things technologies, remote sensing, and environmental sensing systems enable continuous collection of temperature, precipitation, soil moisture, vegetation conditions, and other observations, providing a new basis for disaster identification and environmental exposure characterization. By linking environmental observations with borrower attributes, loan information, and historical repayment records, agricultural credit risk can be characterized from both environmental and borrower perspectives [5]. However, environmental and financial data differ substantially in temporal resolution, spatial scale, and structure, making effective heterogeneous data integration and latent interaction modeling key challenges [6].

Existing agricultural loan default prediction methods mainly rely on borrower attributes and historical repayment records [7]. Logistic regression and decision trees provide favorable interpretability but depend heavily on handcrafted features and have limited ability to capture complex nonlinear relationships [8]. Random forests, support vector machines, and gradient boosting models improve risk identification but remain limited in automatically representing heterogeneous multimodal information [9]. When environmental observations are introduced, simple feature concatenation cannot fully capture interactions between environmental exposure and borrower characteristics [10]. For example, the same heat event may affect borrowers with different operational scales and production patterns differently, which is difficult to represent using conventional approaches [11].

Extreme climatic events are also low-frequency but high-impact, resulting in limited real disaster samples and insufficient representation of extreme-risk patterns [12]. Furthermore, empirical observations in agricultural credit research are inevitably constrained by operational and geographic boundary conditions. In the present study, the primary empirical investigations focus on representative agricultural counties in Shijiazhuang and Handan within Hebei Province, situated in the North China Plain, which is characterized by a temperate semi-arid monsoon climate and major cropping patterns spanning winter wheat, summer maize, greenhouse vegetables, and cotton. Understanding such regional cropping and geographic boundaries early is vital for establishing the operational context and transferability limits of sensing-driven credit risk models. Unconstrained augmentation may generate unrealistic environmental combinations and reduce model reliability [13]. Environmental sensing systems are further affected by missing observations, measurement errors, and abnormal fluctuations, which may compromise training stability and predictive reliability [14]. Consequently, agricultural credit risk prediction under extreme climates requires both high predictive performance and robustness to distributional variation, degraded sensing, and geographic shifts [15].

Deep learning has been widely applied to agricultural intelligence and financial risk prediction because of its strong nonlinear representation capability [16]. Convolutional neural networks, recurrent neural networks, and Transformer-based architectures can automatically learn latent representations from high-dimensional data and facilitate the integration of environmental observations with borrower information [17]. Attention mechanisms are particularly suitable for capturing dependencies among heterogeneous variables. However, existing deep learning methods still focus mainly on predictive performance, with insufficient consideration of extreme-sample scarcity, input uncertainty, and external reliability [18]. A robust framework integrating environmental sensing, extreme-risk learning, and predictive uncertainty is therefore required [19]. Wu et al. [2] proposed a Multimodal Credit Risk Modelling Framework integrating structured credit variables, climate panel data, and text through LSTM-, GRU-, and Transformer-based models, and demonstrated the importance of climate-related flood risk through SHAP analysis. Wang et al. [4] developed a Multi-Source Remote Sensing Digital Footprint Risk Profiling Framework that combined remotely sensed environmental characteristics with agricultural borrower information, demonstrating the value of remote sensing for agricultural credit risk identification. Abbas et al. [20] proposed an Interpretable Hybrid Stacking Model that combined multiple machine learning classifiers for multiclass loan default prediction and improved decision transparency through model interpretation. Notwithstanding these advances, existing benchmarks, including the Previous Model benchmark [4], predominantly rely on static feature concatenation or unweighted optimization objectives, falling short in capturing dynamic bi-directional feature interactions, mitigating data corruption, or explaining loan-level decisions under extreme environmental shocks.

To address these challenges, a robust agricultural loan default prediction framework integrating sensing observations, borrower characteristics, and uncertainty-aware learning under extreme climatic conditions is proposed. Agricultural environmental observations and borrower information are first represented through separate encoding branches, followed by cross-attention to model interactions between environmental exposure and borrower behavior. A climate-constrained disaster scenario augmentation strategy is then introduced to expand the training space under regional, seasonal, and inter-variable constraints with rigorous label assignment rules. Predictive uncertainty is further estimated and converted into stabilized sample weights to reduce the influence of highly uncertain samples and anomalous observations. Model generalizability and reliability are evaluated under retrospective real disaster events (heatwaves and floods), systematic sensing degradation settings, as well as external temporal (holdout future cohort) and cross-regional splits, while prospective institutional pilot deployment is identified as a critical future operational direction.

The main contributions of this study are summarized as follows:

1. A cross-source interaction representation approach integrating agricultural environmental observations and borrower credit information is proposed. A dual-branch Transformer encoding architecture and bidirectional cross-attention mechanism are used to capture associations between environmental exposure and borrower attributes, enabling the incremental predictive value of sensing observations to be systematically evaluated while bounding feature representations to prevent unconstrained amplification.
2. A climate-constrained disaster scenario augmentation approach with explicit physical boundaries and reproducible labeling criteria is proposed. Regional conditions, seasonal characteristics, and dependencies among environmental variables are used to restrict the augmentation space, ensuring that generated scenarios remain consistent with realistic agricultural environmental dynamics while separating supervised training variants from unverified stress-testing counterfactuals.
3. An uncertainty-guided stabilized learning mechanism is developed. Weight normalization, upper-bound clipping, and gradient detachment are introduced into the uncertainty weighting process to suppress the influence of extreme sample weights and improve the stability and calibration of probabilistic risk prediction under observation noise and label ambiguity.
4. A comprehensive validation framework oriented toward practical deployment conditions is established. Observation missingness, measurement errors, spatial matching sensitivity, synthetic labeling variations, retrospective real disaster events, and external temporal and cross-regional validation are systematically evaluated to characterize model applicability, operational boundaries, and explainability via Shapley additive explanations.

## 2. Related Work

### 2.1. Agricultural Environmental Sensing and Cross-Source Data Fusion

The development of agricultural environmental sensing technologies has provided a new data basis for monitoring production conditions and conducting agricultural risk analysis [21]. Traditional agricultural management has mainly relied on manual surveys, historical statistics, and regional meteorological records, which are often insufficient for characterizing dynamic microenvironmental variations experienced by individual farming entities [22]. With advances in Internet of Things sensors, remote sensing, and high-spatiotemporal-resolution meteorological products, agricultural environmental information acquisition has gradually shifted from discrete statistics toward continuous sensing [23]. Current monitoring systems mainly obtain environmental information from ground-based sensors, satellite remote sensing platforms, and meteorological reanalysis products [24]. Ground sensors provide field-scale measurements such as temperature, humidity, soil moisture, and solar radiation; remote sensing supports regional monitoring through vegetation indices, land surface temperature, and land-cover information; and meteorological products provide long-term environmental backgrounds for analyzing climatic trends and extreme events. Environmental sensing data are particularly valuable for characterizing agricultural exposure. Extreme-heat duration reflects heat stress, precipitation anomalies indicate drought-related risks, and soil moisture variations characterize water stress [25]. Incorporating such observations into agricultural risk prediction therefore extends conventional borrower-centered credit assessment toward joint borrower–environment analysis.

However, substantial heterogeneity exists between agricultural environmental observations and conventional financial data [26]. Environmental data are typically continuous time series containing trends, periodic fluctuations, and abrupt anomalies, whereas loan records and credit histories are mainly structured tabular data with lower update frequencies [27]. Environmental observations are also spatially dependent, with impacts varying across regions, fields, and production systems, while credit data are organized around individual borrowers [28]. Effective association of heterogeneous data across different spatiotemporal scales therefore remains a major challenge [29].

Cross-source data fusion has consequently become an important direction in multimodal artificial intelligence. Conventional strategies generally concatenate standardized features from different sources before prediction [30]. Although simple, this approach cannot adequately capture deep inter-source relationships. For example, the same heat event may affect large-scale and small-scale agricultural operators differently, which cannot be fully represented when environmental and borrower variables are treated independently [31]. Representation-learning-based fusion has therefore been increasingly adopted [32]. Dual-branch encoders can separately learn modality-specific representations and integrate them through attention, interaction layers, or joint embeddings. Compared with direct concatenation, such methods better capture nonlinear relationships between environmental conditions and borrower characteristics, supporting more effective agricultural credit risk modeling under extreme climates [33]. Accordingly, agricultural environmental observations are treated as an important source of risk-exposure information, and cross-source interaction representations are constructed through separate environmental and borrower encoders to characterize the association between environmental conditions and agricultural loan default risk.

### 2.2. Default Prediction Driven by Environmental Exposure and Borrower Behavior

Agricultural credit default prediction aims to estimate future default probability from information available during the loan cycle. Conventional credit risk modeling mainly focuses on repayment capacity, credit history, and financial behavior. Variables such as loan amount, repayment history, operational scale, asset status, and income have therefore been widely used in credit scoring [34]. Although these approaches established the basic framework of financial risk prediction, external environmental effects are often treated as secondary. Unlike conventional commercial lending, agricultural production is highly dependent on natural conditions [35]. Agricultural income is closely related to crop yield, production costs, and market conditions, all of which can be affected by temperature, precipitation, drought, and disaster events [36]. Environmental exposure has therefore received increasing attention. Meteorological anomalies, disaster frequency, remotely sensed vegetation changes, and regional production conditions can help quantify the extent to which farming entities are affected by natural hazards [37]. Existing studies have shown that environmental factors can complement conventional credit variables and improve agricultural risk identification [38].

Nevertheless, limitations remain in environmentally driven agricultural risk prediction [39]. Regional-scale environmental indicators are often used as aggregate explanatory variables, while borrower heterogeneity associated with operational patterns, resource endowments, and adaptive capacity is overlooked [40]. In practice, extreme climate events affect agricultural entities differently, and risk transmission often follows the chain of environmental shock, production disruption, income deterioration, reduced repayment capacity, and increased default risk [41]. Joint modeling of environmental exposure and borrower behavior is therefore required [42]. Existing studies also rely heavily on manually designed features and simple combinations [43]. Environmental indicators such as temperature anomalies and precipitation changes are commonly concatenated with credit variables before model training. Although environmental information is introduced, this strategy provides limited capability for representing heterogeneous borrower responses [44]. Deep learning has enabled more automatic interaction modeling; for example, through recurrent networks for meteorological sequences and attention mechanisms for variable importance learning [45]. However, research on agricultural credit risk under extreme climates remains limited, particularly for unified frameworks that jointly consider environmental exposure, borrower vulnerability, and data uncertainty [46]. Accordingly, agricultural environmental sensing information is incorporated into agricultural credit risk prediction through cross-source interaction learning, with emphasis placed on how environmental changes alter borrower risk under extreme climatic conditions.

### 2.3. Uncertainty-Aware and Robust Prediction Methods

Data uncertainty is an important factor affecting model reliability in practical agricultural risk prediction. Agricultural environmental observations are frequently affected by sensor errors, missing values, insufficient spatial coverage, and limited extreme-event samples. Agricultural credit default itself is also relatively infrequent, making complex risk patterns difficult to learn. Predictive accuracy alone is therefore insufficient, and greater attention has been directed toward prediction reliability and stability under previously unseen conditions [47].

A variety of robust prediction strategies have been developed to address these issues. Data augmentation expands the training sample space and improves adaptability to complex scenarios. Random perturbation, generative modeling, and adversarial augmentation have been widely used in other domains, but unconstrained augmentation in agricultural risk prediction may generate environmentally unrealistic combinations. For example, arbitrary changes in temperature and precipitation may violate regional climatic patterns. Augmentation under extreme agricultural conditions should therefore incorporate regional climate, seasonal patterns, and inter-variable dependencies to preserve physical and agronomic plausibility [48].

Based on these developments, an uncertainty-aware mechanism is incorporated into agricultural credit risk prediction. Predictive uncertainty is used to adjust sample-specific training weights, while climate-constrained augmentation improves adaptability to extreme environments and distributional variation. The resulting framework therefore focuses not only on discrimination performance but also on maintaining stable and reliable risk assessment under degraded sensing quality, extreme climatic conditions, and external distribution shifts. Furthermore, to ensure transparent credit decision-making, explainable artificial intelligence (XAI) methodologies, such as Shapley additive explanations (SHAP) [49], are integrated to provide global and localized feature attribution, bridging the gap between deep representation routing and verifiable financial risk governance.

## 3. Materials and Method

### 3.1. Data Collection and Operational Boundary Conditions

A multi-source dataset integrating agricultural environmental sensing observations, vegetation remote sensing information, borrower attributes, and historical repayment records was constructed, as shown in Table 1. Data were collected from representative agricultural counties in Shijiazhuang and Handan, Hebei Province, China, spanning an area located in the North China Plain between latitude $36^{\circ }04^{\prime }\;N$–$38^{\circ }58^{\prime }\;N$ and longitude $113^{\circ }48^{\prime }\;E$–$115^{\circ }29^{\prime }\;E$. This study region is governed by a temperate semi-arid continental monsoon climate, featuring hot, humid summers with concentrated precipitation and cold, dry winters. Agricultural production in this domain is dominated by double-cropping grain systems (winter wheat and summer maize), open-field and facility vegetables, and cash crops (predominantly cotton and fruit). Production is regularly subjected to seasonal extreme climatic hazards, including summer heatwaves, mid-season droughts, and episodic heavy convective precipitation. Highlighting these geographic and crop boundary conditions early is essential, as the empirical conclusions directly reflect this agro-ecological context and establish the baseline domain for subsequent transferability assessments.

The data collection period extended from January 2022 to December 2025, thereby covering both normal climatic conditions and multiple representative extreme weather events. According to the spatial distribution of major agricultural production areas, cultivated fields, and agricultural loan borrowers, 18 agricultural environmental monitoring stations were deployed across the study region. Air temperature and relative humidity were measured using Vaisala HMP155 sensors (Vaisala Oyj, Vantaa, Finland), precipitation was recorded using ARG100 rain gauges (Environmental Measurements Ltd., North Shields, UK), and solar radiation was monitored using Apogee SP-510 sensors (Apogee Instruments, Inc., Logan, UT, USA). The raw sampling interval for ground-based environmental observations was uniformly set to 10 min. Time synchronization, local buffering, and periodic data transmission were performed using CR1000X data loggers (Campbell Scientific, Inc., Logan, UT, USA).

Soil environmental information was primarily collected from representative farmland associated with the agricultural production activities of loan borrowers. Decagon 5TE probes (Decagon Devices, Inc., Pullman, WA, USA) were installed at soil depths of 10, 30, and 50 cm within each monitoring field to continuously record volumetric soil water content ($\theta_{v}$, in $m^{3}/m^{3}$), soil temperature ($T_{\mathrm{soil}}$, in °C), and electrical conductivity, thereby characterizing root-zone moisture conditions as well as persistent drought or excessive soil wetness.

Following sensor quality-flag inspection, anomaly identification, and missing-data processing, the original high-frequency measurements were aggregated into hourly and daily features. Anomaly identification was conducted by calculating the standardized deviation $z_{t}=\left(x_{t}-\mu_{x}\right)/\sigma_{x}$ over a 30-day historical rolling window (comprising 4320 10-min intervals). Observations with $|z_{t}|>3.0$ that were flagged by hardware error codes or exhibited physically implausible temporal jumps (e.g., rate of temperature change exceeding 10°C over 10 min, or frozen sensor values persisting unchanged for more than 6 consecutive intervals) were designated as measurement anomalies. For anomalous or missing segments shorter than or equal to 2 h, Piecewise Cubic Hermite Interpolating Polynomial (PCHIP) interpolation was applied to preserve local monotonic trends without overshoot; isolated missing single timestamps were linearly interpolated; gaps exceeding 6 h were flagged as missing observation intervals and handled through mask representations. Genuine meteorological extremes (such as persistent heatwaves exceeding 38°C verified by neighboring station records) were strictly retained.

The derived environmental indicators include daily maximum temperature ($T_{\max}$, °C), daily cumulative precipitation (*R*, mm), solar radiation (*S*, ${W/m}^{2}$), consecutive high-temperature days (defined using a high-temperature threshold of $T_{h}=35\;{}^{\circ}C$), consecutive rain-free days, cumulative precipitation anomaly, root-zone soil moisture anomaly, and drought and flood intensity indicators. Formally, the cumulative precipitation anomaly $P_{i}$ and standardized soil moisture anomaly $SMA_{i}$ over a time window *T* are expressed as:

(1) $$P_{i}=\sum_{t=1}^{T}R_{i}\left(t\right)-{\bar{R}}_{i},\;SMA_{i}\left(t\right)=\frac{\theta_{i}\left(t\right)-{\bar{\theta }}_{i}}{\sigma_{\theta ,i}},\;$$

where ${\bar{R}}_{i}$ is the historical multi-year mean precipitation for the corresponding calendar window, and ${\bar{\theta }}_{i}$ and $\sigma_{\theta ,i}$ are the historical mean and standard deviation of volumetric soil water content at the 30 cm root-zone depth. The drought intensity indicator $DII_{i}$ and flood exposure intensity indicator $FII_{i}$ are formulated as:

(2) $$DII_{i}=\sum_{t=1}^{T}\max\left(0,-SMA_{i}\left(t\right)\right)\cdot I\left(R_{i}\left(t\right)<0.1\;\mathrm{mm}\right),\;FII_{i}=\sum_{t=1}^{T}\max\left(0,R_{i}\left(t\right)-R_{95\mathrm{th}}\right),$$

where I(·) is the indicator function, and $R_{95\mathrm{th}}$ represents the regional 95th percentile threshold of daily precipitation.

To improve the spatial coverage of environmental information, Sentinel-2 MSI multispectral remote sensing imagery covering the study region was acquired from the European Space Agency (Paris, France) during the same period. Cloud masking, spatial registration, and field-level cropping were performed according to the actual agricultural operating boundaries of loan borrowers. The Normalized Difference Vegetation Index (NDVI), Enhanced Vegetation Index (EVI), and vegetation-condition variation features were subsequently extracted, resulting in a total of 8436 valid field–date remote sensing records. These field-level multispectral observations provide 10m to 20m spatial resolution, effectively mitigating plot-level microclimate discrepancies between ground monitoring stations and actual farmland parcels.

Agricultural borrower and loan-related data were obtained from anonymized business databases provided by cooperating financial institutions operating in the study region, with the collection period aligned with that of the environmental observations. A total of 12,684 valid loan samples were retained. The borrower input vector comprises 18 structured variables spanning demographic characteristics (age, education), operational scale (cultivated land area in hectares, years of continuous agricultural operation), agricultural endowment (crop category, farmland irrigation infrastructure status encoded as a binary indicator for fully irrigated versus rainfed plots, agricultural machinery asset valuation), loan contractual terms (loan amount, interest rate, repayment schedule, loan term), credit history (prior loan count, historical overdue frequency, internal credit rating grade), and financial leverage (debt-to-income ratio, operating cash flow margin). Categorical variables were converted using defined encoding schemes: One-Hot Encoding was applied to crop categories (grain, vegetable, cotton/cash crops); Ordinal Encoding was applied to internal credit rating grades and education tiers; and Frequency Encoding was utilized for administrative county identifiers.

Default labels were determined according to contractual loan obligations and subsequent actual repayment outcomes, defined as non-payment exceeding 90 days past due. The prediction time point and default observation window were strictly separated to prevent post-outcome information from entering the input features. To link borrower operating entities with environmental conditions, a multi-station distance-decay weighting scheme across the N=3 nearest environmental monitoring stations within a 35km operational radius was implemented (as formulated in Equations (3) and (4)), while the corresponding field parcel was directly linked to Sentinel-2 remote sensing pixels. Environmental observation sequences covering the preceding 30, 60, and 90 d were extracted relative to each prediction time point, forming a multi-source risk prediction dataset where environmental exposure, borrower characteristics, historical repayment information, and default labels were aligned.

### 3.2. Data Preprocessing and Climate-Constrained Augmentation Formulation

Agricultural environmental observations and agricultural credit data are obtained from heterogeneous sources and are commonly affected by measurement errors, missing records, inconsistent temporal resolutions, and differences in spatial scale. Directly feeding raw data into predictive models may cause spurious data patterns to be learned, thereby reducing the reliability of risk prediction. Therefore, multi-source data are subjected to quality control, spatiotemporal matching, feature construction, and standardization before model training. In addition, because extreme climatic events are characterized by low occurrence frequency but potentially severe impacts, the number of real disaster samples is generally limited. Augmented samples are therefore constructed under constraints consistent with agricultural environmental processes to improve the capability of the model to learn extreme-risk states.

For spatial matching, entity *i* at time *t* is linked to monitoring stations through multi-station inverse distance weighting across the N=3 nearest stations:

(3) $$E_{i}\left(t\right)=\frac{\sum_{j=1}^{N}w_{ij}E_{j}\left(t\right)}{\sum_{j=1}^{N}w_{ij}},$$

where $E_{i}\left(t\right)$ denotes the fused environmental feature associated with entity *i*, $E_{j}\left(t\right)$ denotes the observation at the *j*th monitoring station, $w_{ij}$ denotes the spatial decay weight, and the weight decay function is given by:

(4) $$w_{ij}=\frac{1}{d_{ij}^{\alpha }},$$

where $d_{ij}$ is the Euclidean distance in kilometers between entity *i*’s agricultural parcel and monitoring station *j*, and α is the distance-decay coefficient (fixed to α=1.0 as default based on spatial decay principles and validation tuning). When comparing matching schemes, single nearest-station matching corresponds to setting N=1.

Continuous variables are standardized using *z*-score normalization:

(5) $$x^{\prime }=\frac{x-\mu }{\sigma },\;$$

where *x* denotes the original feature, and μ and σ denote the mean and standard deviation estimated exclusively from the training set, respectively. Categorical variables are transformed using the aforementioned encoding schemes.

To prevent data leakage and ensure reliable model evaluation, the development and test sets are partitioned according to borrower entity grouping. The test set remains completely independent and is strictly excluded from all preprocessing-parameter estimation, augmentation design, and model selection. All standardization parameters, missing-data handling rules, and augmentation parameters are estimated exclusively from the training data.

During model training, scenario augmentation generates extreme-risk samples under physical and agronomic constraints: $X_{aug}=G\left(X,\theta_{c},\theta_{s},\theta_{v}\right)$, where $\theta_{c}$, $\theta_{s}$, and $\theta_{v}$ represent regional climatic, seasonal, and inter-variable relationship constraints, respectively. Crucially, the augmented dataset is organized into three distinct categories with reproducible criteria and labeling thresholds:

Category 1: Supervised Climate-Constrained Disaster Samples ($X_{clim}^{+}$). When synthetic disaster scenarios are derived by perturbing observed extreme events within a bounded neighborhood ($∥\Delta_{clim}∥\;\leq 1.5\sigma$, where σ represents the empirical standard deviation of the corresponding climate feature distribution), the borrower’s operational status, asset endowment, and debt obligations remain fundamentally consistent. Under this bounded perturbation threshold, the underlying loan solvency outcome is preserved, and the augmented sample inherits the ground-truth default label $y_{i}$. These samples are included in supervised training batches.

Category 2: Supervised Sensing-Degraded Samples ($X_{obs}^{+}$). Samples generated by injecting observation missingness masks, Gaussian measurement noise ($\sigma_{\eta }\leq 0.5$), and systematic calibration drift (≤30%) represent corruptions of the measurement apparatus rather than changes in the borrower’s actual economic outcome. Consequently, these samples strictly inherit the original default label $y_{i}$ and are included in supervised training batches.

Category 3: Unsupervised Counterfactual Stress-Testing Scenarios. Scenarios generated under compounding, out-of-distribution shocks ($∥\Delta_{clim}∥\;>1.5\sigma$, or hypothetical multi-hazard events exceeding the historical 100-year return envelope) lack verifiable default outcomes. Therefore, to prevent introducing spurious training bias, all Category 3 counterfactual samples are strictly excluded from supervised training and reserved exclusively for post-hoc stress testing and sensitivity evaluation.

The supervised augmentation rate during training was set to 30% of the original training set size (7547 loans), allocated equally as 15% Category 1 climate-scenario samples and 15% Category 2 sensing-degraded samples (amounting to 1132 samples in each category, or 2264 augmented training samples in total, representing an effective augmentation rate of 2264/7547≈29.9987%≈30%, yielding 9811 total training samples).

Furthermore, candidate scenarios generated by $G_{\theta }$ are rejected if they violate the domain rules Ω(c): (1) Temperature must reside within regional historical bounds [−20°C,45°C]; (2) Daily precipitation R≥0mm and relative humidity RH∈[0%,100%]; (3) Physical vapor pressure consistency requires R=0mm when RH<20%; (4) Seasonal constraints disallow freezing temperatures (T<0°C) between June and August and heatwave temperatures (T>35°C) between December and February; (5) Agronomic root-zone volumetric soil moisture is strictly bounded within $\left[0.05\;m^{3}/m^{3},0.45\;m^{3}/m^{3}\right]$ (from permanent wilting point to field saturation).

### 3.3. Proposed Method

#### 3.3.1. Overall Framework Architecture

The uniformly organized environmental observation sequences and agricultural loan borrower characteristics are first processed through a dual-branch encoding architecture. In the environmental observation branch, temporal feature mapping, local pattern extraction, and deep representation learning are successively performed to obtain representations of climatic conditions and disaster exposure. In parallel, operational attributes, loan information, and historical repayment behavior are embedded and transformed through nonlinear mappings in the borrower feature branch to construct borrower-specific risk representations. The two latent representations are subsequently introduced into a bidirectional cross-attention module. Environmental representations are used to dynamically modulate the importance of borrower risk characteristics, while borrower representations inversely condition the interpretation of environmental exposure, thereby producing a joint risk representation capable of characterizing the effects of specific environmental shocks on heterogeneous borrowers. During training, while the original samples are propagated through the primary prediction network, a climate-constrained disaster scenario augmentation module generates plausible extreme-environment variants according to regional climatic backgrounds, seasonal conditions, crop growth stages, disaster types, and inter-variable dependencies. Observation-degradation samples involving sensor missingness, measurement noise, and systematic bias are independently generated and are combined with the original samples after feasibility and consistency screening. The resulting joint risk representations are then passed to a risk prediction network equipped with stochastic dropout. Multiple stochastic forward passes are performed to obtain a set of default probability estimates, whose mean is used as the final risk prediction, while their dispersion is used to characterize predictive uncertainty. The estimated uncertainty is subsequently transformed into sample-specific learning weights, followed by within-batch normalization, upper-bound clipping, and gradient detachment to prevent noisy or highly uncertain samples from exerting excessive influence on parameter optimization. Finally, the stabilized weights are incorporated into the default classification loss, together with a prediction-consistency constraint between original and observation-perturbed samples. This end-to-end optimization enables the environmental encoder, borrower encoder, cross-source interaction layer, and risk prediction head to be jointly updated, while simultaneously producing the agricultural loan default probability and its associated predictive uncertainty.

#### 3.3.2. Environmental Observation–Borrower Feature Interaction Representation

**Figure 1:**
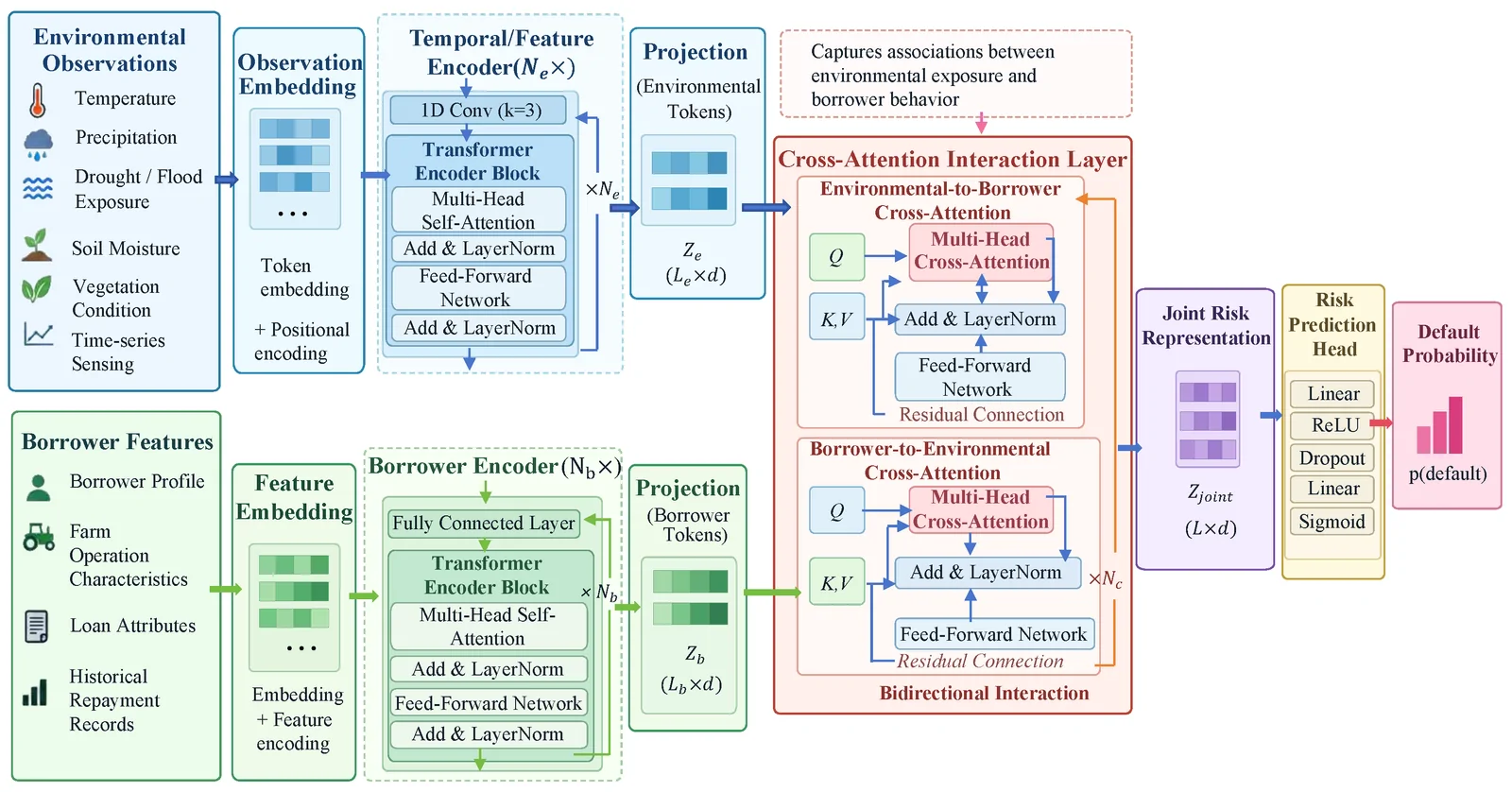
Cross-source interaction between environmental observations and borrower features is achieved through dual-branch encoding and bidirectional cross-attention to generate a joint risk representation for default probability prediction.

The first module adopts parallel environmental observation and borrower feature branches, followed by bidirectional cross-attention for cross-source risk interaction, fully aligning the text with the structural layout depicted in Figure 1. The environmental input is organized as a temporal sequence tensor $X_{e}\in R^{L_{e}\times C_{e}}$, where $L_{e}=90$ represents the 90-day historical observation sequence and $C_{e}=12$ represents the environmental features (including temperature, precipitation, humidity, solar radiation, soil moisture, and remote sensing indices). The environmental encoder begins with a one-dimensional convolutional layer (kernel size $K_{e}=3$, stride 1, padding 1) that projects the raw channels from $C_{e}$ to the latent embedding dimension $D_{e}=128$. Sinusoidal positional encodings are added to preserve sequential temporal ordering, after which the sequence is processed by $N_{e}=3$ consecutive Transformer encoder blocks. Each Transformer block contains a multi-head self-attention layer with $h_{e}=4$ heads (key/value dimension $d_{k}=32$), LayerNorm, GeLU nonlinear activation, a feed-forward network expanding intermediate channels to 256 before projecting back to 128, a dropout rate of 0.3, and residual skip connections.

In parallel, the borrower input $X_{b}\in R^{C_{b}}$ ($C_{b}=18$ structured attributes) is mapped via an initial linear transformation into a set of feature token embeddings of dimension $D_{b}=128$, supplemented by learnable feature-type embeddings. The resulting tokens are fed into $N_{b}=2$ borrower Transformer encoder blocks, each equipped with multi-head self-attention ($h_{b}=4$ heads, key/value dimension 32), LayerNorm, GeLU activation, feed-forward expansion to 256, dropout rate 0.3, and residual connections, producing the borrower latent representation $B\in R^{S_{b}\times D}$ where D=128.

As shown in Figure 1, the two representations are projected into a common attention space of dimension D=128:

(6) $$E=\phi_{e}\left(X_{e}\right)W_{e}^{p}+b_{e}^{p},\;B=\phi_{b}\left(X_{b}\right)W_{b}^{p}+b_{b}^{p},$$

where $\phi_{e}\left(\cdot \right)$ and $\phi_{b}\left(\cdot \right)$ denote the environmental encoding network and borrower encoding network, respectively, while E and B denote their latent representations after dimensional alignment. A total of M=4 attention heads are subsequently constructed, with each head learning the conditional contribution of environmental information to borrower-specific risk:

(7) $$A_{b\leftarrow e}^{\left(m\right)}=\mathrm{Softmax}\left(\frac{\left(BW_{Q}^{\left(m\right)}\right){\left(EW_{K}^{\left(m\right)}\right)}^{T}}{\sqrt{d_{m}}}\right),$$

and the corresponding environmental value vectors are aggregated as

(8) $$R_{b\leftarrow e}^{\left(m\right)}=A_{b\leftarrow e}^{\left(m\right)}EW_{V}^{\left(m\right)}.$$

For the reverse interaction, the environmental representation is used as the query, whereas the borrower representation is used as both the key and value, resulting in $R_{e\leftarrow b}^{\left(m\right)}$. After the outputs of multiple attention heads are concatenated along the channel dimension, bidirectional interaction representations are generated through output projection, residual connections, and a feed-forward network:

(9) $$Z=\psi \left(\left[{\mathrm{Concat}}_{m=1}^{M}R_{b\leftarrow e}^{\left(m\right)}\;;{\mathrm{Concat}}_{m=1}^{M}R_{e\leftarrow b}^{\left(m\right)}\right]W_{o}\right),$$

where ψ(·) denotes the interaction fusion network consisting of normalization, feed-forward transformation, and residual mapping. Its internal feature width is expanded from D=128 to $D_{f}=256$ and subsequently compressed back to D=128, while the interaction layer is stacked $N_{c}=2$ times. A fixed-length joint risk vector is finally obtained through attention pooling:

(10) $$z=\sum_{j}\frac{\exp(q^{T}Z_{j})}{\sum_{k}\exp\left(q^{T}Z_{k}\right)}Z_{j}.$$

The theoretical validity of this design can be explained by the normalization property of the attention weights. Because the Softmax output satisfies

(11) $$A_{ij}\geq 0,\;\sum_{j}A_{ij}=1,\;$$

each interaction representation constitutes a convex combination of latent features from the complementary data source. Accordingly, its norm satisfies

(12) $$∥\sum_{j}A_{ij}v_{j}∥\leq \sum_{j}A_{ij}∥v_{j}∥\leq \max_{j}∥v_{j}∥.\;$$

Therefore, cross-attention does not cause unconstrained amplification of feature magnitudes while introducing cross-source information, which contributes to stable model optimization. Compared with direct feature concatenation, bidirectional interaction enables temperature, precipitation, soil moisture, and disaster-exposure information to be dynamically selected according to borrower-specific operational characteristics, while environmental conditions can simultaneously modulate the importance of different borrower attributes. This property is particularly suitable for agricultural loan default prediction, where the impact of extreme climatic events is highly heterogeneous across borrowers.

#### 3.3.3. Climate-Constrained Disaster Scenario Augmentation

This module takes an environmental observation tensor $X\in R^{H\times W\times C}$ as input, where *H* denotes the temporal dimension, *W* denotes the number of environmental variables, and *C* denotes the channels corresponding to raw observations and derived features. Regional information, season, crop growth stage, and disaster type are simultaneously encoded into a conditional vector. The condition encoder consists of $N_{c}=2$ fully connected layers, whose hidden width is progressively mapped from $D_{c}$ to a unified latent dimension D=128. The environmental tensor is first processed by a one-dimensional convolution with kernel width $K_{g}=3$, through which the channel dimension is expanded from *C* to $C_{g}=128$, and is subsequently passed through a scenario generator composed of $N_{g}=3$ residual generation blocks.

**Figure 2:**
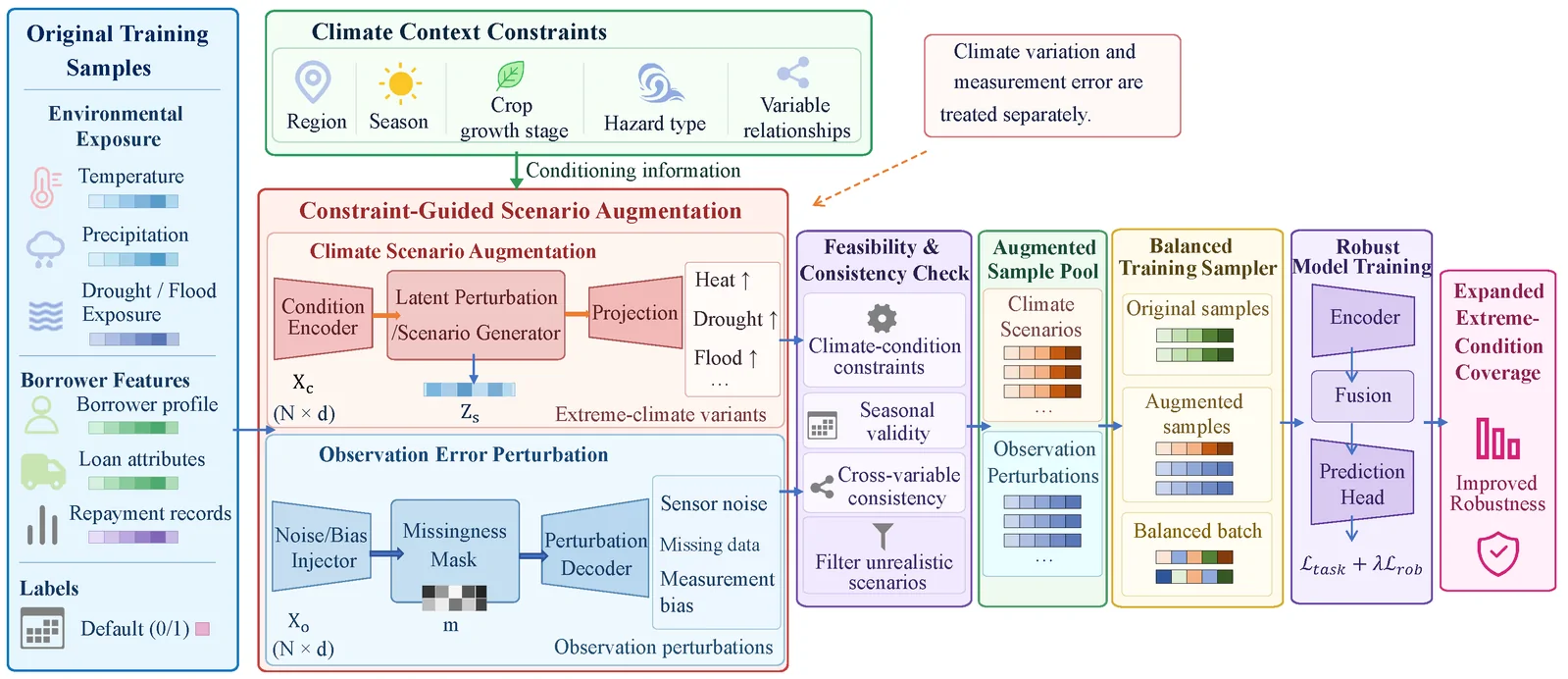
The climate-constrained disaster scenario augmentation framework expands training samples through climate-scenario generation and observation-error perturbation, followed by feasibility and consistency screening to improve robustness under extreme conditions.

As shown in Figure 2, each generation block contains convolution, LayerNorm, nonlinear activation, and conditional modulation, while the output spatial dimensions are maintained at H×W. The conditional modulation process is formulated as

(13) $$F_{l}^{*}=\gamma_{l}\left(c\right)⊙\mathrm{LN}\left(F_{l}\right)+\beta_{l}\left(c\right),$$

where $\gamma_{l}\left(\cdot \right)$ and $\beta_{l}\left(\cdot \right)$ adaptively modulate the features of the *l*th layer according to the climatic condition vector. The generator subsequently produces a disaster perturbation field:

(14) $$\Delta_{clim}=s\cdot \tanh\left(G_{\theta }\left(X,c\right)\right),$$

where s=0.5 denotes the augmentation-intensity control factor, and tanh(·) constrains the perturbation magnitude. The candidate scenario is then projected into a climate-feasible domain:

(15) $$X_{clim}^{+}=\Pi_{\Omega (c)}\left(X+\Delta_{clim}\right),$$

where Ω(c) is jointly determined by regional climatic ranges, seasonal boundaries, disaster-direction constraints, and relationships among variables such as temperature, precipitation, and soil moisture, thereby filtering physically implausible combinations. When these constraints are relaxed to form a closed convex feasible set, the Euclidean projection satisfies the non-expansive property:

(16) $${∥\Pi_{\Omega }\left(a\right)-\Pi_{\Omega }\left(b\right)∥}_{2}\leq {∥a-b∥}_{2},$$

which demonstrates that constraint-based projection does not amplify the perturbation distance between candidate samples and therefore helps suppress distributional drift caused by excessive augmentation. In parallel, observation-quality degradation is modeled through an independent network branch, in which the underlying climatic state remains unchanged. A total of $N_{o}=2$ perturbation blocks are used to generate noise, bias, and missingness masks:

(17) $$X_{obs}^{+}=M⊙\left(X+\eta N_{\phi }\left(X\right)+B_{\phi }\left(X\right)\right),\;$$

where M controls random or continuous missingness, while $N_{\phi }\left(\cdot \right)$ and $B_{\phi }\left(\cdot \right)$ characterize measurement noise and systematic bias, respectively. After consistency screening, both categories of augmented samples are combined with the original samples to form the training batches. Consequently, variations in risk-related characteristics under genuine extreme climatic states can be learned while robustness to sensor missingness and measurement errors is simultaneously enhanced. By explicitly separating environmental-state variation from observation degradation, the proposed augmentation strategy expands the coverage of extreme-risk conditions while maintaining realistic dependencies among climatic variables, making it particularly suitable for agricultural loan risk prediction scenarios characterized by scarce extreme events and unstable environmental sensing quality.

#### 3.3.4. Uncertainty-Guided Stabilized Weighted Learning

This module receives the joint risk representation generated by the first module and constructs stabilized sample weights from stochastic prediction outcomes. The input feature tensor is denoted by $Z\in R^{H_{u}\times W_{u}\times C_{u}}$, where $H_{u}$, $W_{u}$, and $C_{u}$ represent the height, width, and channel dimensions of the joint representation, respectively. Global pooling is first applied to compress the tensor into a $D_{0}=128$-dimensional risk vector, which is subsequently passed through a prediction head composed of $N_{p}=2$ layers. The width of the *l*th layer is denoted by $D_{l}$ ($D_{1}=64,D_{2}=1$), and linear transformation, ReLU activation, Dropout, and normalization are successively applied to progressively compress the feature representation. The Dropout retention rate of the *l*th layer is denoted by $q_{l}$ (dropout probability 0.3), and the final output is a single-channel default-risk score.

**Figure 3:**
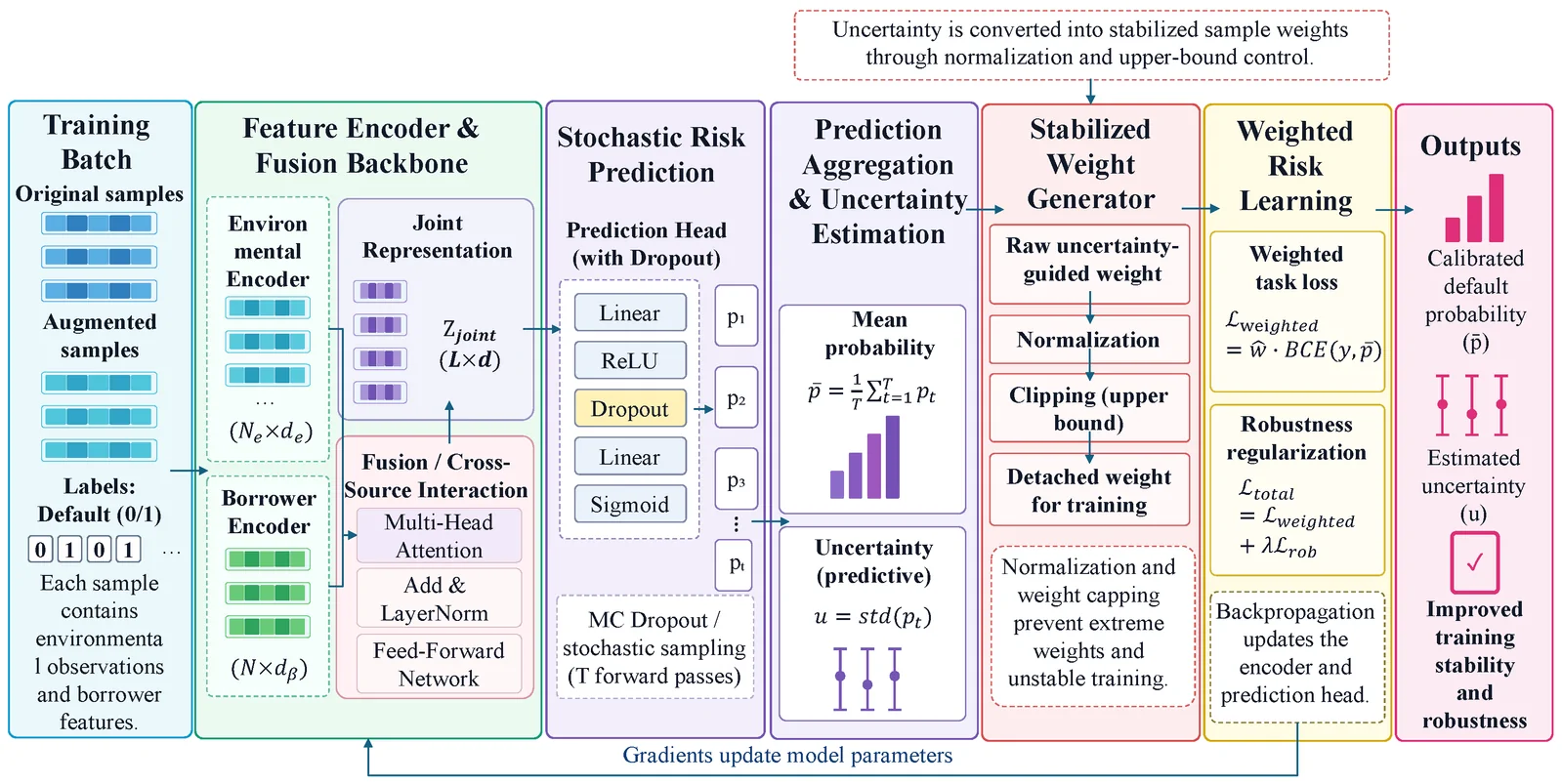
The uncertainty-guided stabilized weighted learning framework improves the stability and reliability of default probability prediction through stochastic risk prediction, uncertainty estimation, weight normalization and clipping, and weighted loss optimization.

As shown in Figure 3, stochastic dropout remains active during prediction, and T=20 stochastic forward passes are performed for the same sample. The prediction obtained from the *t*th pass is expressed as

(18) $$p_{i}^{\left[t\right]}=\sigma \left(W_{p}F_{\theta }\left(z_{i};\xi^{\left[t\right]}\right)+b_{p}\right),$$

where $\xi^{\left[t\right]}$ denotes the stochastic dropout mask used during the *t*th forward pass. To avoid excessive dependence on a single central estimate of variance, pairwise prediction discrepancies are used to quantify model uncertainty:

(19) $$U_{i}={\left[\frac{1}{T(T-1)}\sum_{t=1}^{T}\sum_{\begin{array}{c} s=1\\ s\neq t \end{array}}^{T}{\left(p_{i}^{\left[t\right]}-p_{i}^{\left[s\right]}\right)}^{2}\right]}^{1/2}.$$

The final risk output is aggregated through the expectation of the stochastic prediction set:

(20) $${\hat{p}}_{i}=E_{t}\left[p_{i}^{\left[t\right]}\right].$$

The estimated uncertainty is subsequently mapped to a non-negative raw confidence weight. To reduce the training contribution of highly uncertain samples while avoiding excessively rapid weight decay, a smooth reciprocal transformation is adopted:

(21) $$r_{i}=\frac{1}{\varepsilon +\mathrm{Softplus}\left(\alpha U_{i}\right)},$$

where α=0.5 controls the strength of uncertainty-based weighting, and $\varepsilon =10^{-4}$ ensures numerical stability. Within-batch scale calibration and weight-bound control are subsequently defined as

(22) $$\omega_{i}=\mathrm{clip}\left(\frac{Br_{i}}{\sum_{j=1}^{B}r_{j}},\omega_{\min},\omega_{\max}\right),$$

and $\omega_{i}$ is treated as a constant during backpropagation via gradient detachment $\mathrm{sg}(\omega_{i})$. This operation prevents the network from indirectly manipulating sample weights by deliberately modifying predictive uncertainty. The final optimization objective is defined as

(23) $$J=-\frac{1}{B}\sum_{i=1}^{B}\mathrm{sg}\left(\omega_{i}\right)\left[y_{i}\log{\hat{p}}_{i}+\left(1-y_{i}\right)\log\left(1-{\hat{p}}_{i}\right)\right]+\lambda R_{c},$$

where sg(·) denotes the stop-gradient operator, $R_{c}={∥{\hat{p}}_{i}-{\hat{p}}_{i}^{\mathrm{perturbed}}∥}^{2}$ constrains prediction consistency between original samples and their observation-perturbed counterparts, and λ=0.1. The proposed mechanism provides an explicit stability guarantee. Because the clipping operation satisfies

(24) $$\omega_{\min}\leq \omega_{i}\leq \omega_{\max},$$

with $\omega_{\min}=0.2$ and $\omega_{\max}=2.0$, the contribution of any individual sample to the parameter gradient is bounded. If the gradient norm of the classification loss for a single sample satisfies $∥\nabla_{\theta }\ell_{i}∥\;\leq G$, then

(25) $$∥\nabla_{\theta }\left[\mathrm{sg}\left(\omega_{i}\right)\ell_{i}\right]∥=\omega_{i}∥\nabla_{\theta }\ell_{i}∥\leq \omega_{\max}G.$$

Consequently, even when extreme-climate samples or sensing-degradation samples produce substantial predictive fluctuations, their influence on gradient updates remains bounded, thereby reducing training instability caused by anomalous or highly uncertain observations. At the same time, sufficiently confident samples retain meaningful learning contributions. For agricultural loan default prediction, this mechanism enables default probabilities and associated confidence information to be produced simultaneously while improving training stability, probability reliability, and cross-scenario robustness under extreme climatic conditions, observation missingness, and measurement noise.

### 3.4. Experimental Setup and Baseline Configurations

#### 3.4.1. Hardware, Software, and Leakage-Free Data Partitioning

All experiments were conducted on a dedicated high-performance computing workstation equipped with an Intel Xeon Gold 6330 processor (Intel Corporation, Santa Clara, CA, USA; 2.0 GHz, 28 cores), 128 GB DDR4 ECC RAM, an NVIDIA GeForce RTX 4090 GPU (NVIDIA Corporation, Santa Clara, CA, USA; 24 GB GDDR6X VRAM), and high-speed NVMe solid-state storage. The software environment was built on Ubuntu 22.04 LTS, Python 3.10, PyTorch 2.1.2 with CUDA 12.1 and cuDNN 8.9, Scikit-learn 1.3.2, XGBoost 2.0.3, LightGBM 4.1.0, and Optuna 3.5.0 [50]. The SHAP implementation is documented at https://shap.readthedocs.io/ (accessed on 8 October 2026).

To prevent information leakage between loan records belonging to the same operating entity, a borrower-level grouped partitioning strategy was strictly enforced based on unique borrower identifiers. The core 2022–2024 study cohort (10,782 records, including the internal test partition) was partitioned into a training set (70%, 7547 loans), a validation set (15%, 1617 loans), and an in-distribution test set (15%, 1618 loans). The training set was dedicated to model parameter optimization and augmented sample generation; the validation set was utilized exclusively for hyperparameter optimization via Optuna and early stopping; and the test set was reserved strictly for final out-of-sample evaluation, remaining completely unobserved during preprocessing parameter estimation and tuning. For final performance reporting on the held-out test set, models were trained on the training set and evaluated over five independent runs with distinct random initialization seeds. These five final runs represent repeated evaluations with different stochastic initializations rather than cross-validation folds: the reported confidence intervals summarize variation across independently initialized runs evaluated on the same fixed test cases. An additional set of 1902 loan records originated in 2025 was set aside as an external temporal validation cohort, while county-level geographic holdouts between Shijiazhuang and Handan were utilized for cross-regional transfer evaluation.

Model optimization employed the Adam optimizer with an initial learning rate of $1\times 10^{-3}$, batch size of 64, weight decay of $1\times 10^{-4}$, and a maximum training budget of 100 epochs, coupled with an Early Stopping mechanism terminating training after 20 epochs without improvement on validation PR-AUC.

#### 3.4.2. Baseline Models, Formal Definition of Benchmark, and Hyperparameter Tuning

To rigorously evaluate default risk prediction, nine comparative baselines were implemented: Logistic Regression [51], SVM [52], Random Forest [53], XGBoost [54], LightGBM [55], DNN [56], LSTM [57], Deep Ensemble [58], and the Previous Model benchmark [4].

Hyperparameter tuning for all comparative baselines as well as the proposed framework was systematically conducted on the validation partition using Optuna Bayesian optimization [50] based on the Tree-structured Parzen Estimator (TPE) algorithm. For each model, a uniform search budget of 50 optimization trials was allocated, with the objective function configured to maximize validation PR-AUC, thereby mitigating class-imbalance sensitivity during tuning. Continuous hyperparameters governing model regularization, tree splitting thresholds, and learning rates were sampled from either log-uniform or uniform distributions, while discrete structural parameters (e.g., layer depths, attention head counts, and kernel types) were sampled over predetermined categorical grids. The complete hyperparameter search spaces, sampling distributions, and final optimal configurations for all models are summarized in Table 2.

The resulting optimal architectural and hyperparameter configurations are detailed as follows:

- **Logistic Regression:** $L_{2}$ regularization penalty with inverse regularization parameter C=0.1, solved via L-BFGS.
- **SVM:** Radial basis function (RBF) kernel with penalty parameter C=1.5, gamma set to scale ($1/(N_{\mathrm{features}}\cdot X_{\mathrm{var}})$), and probability calibration via Platt scaling.
- **Random Forest:** 200 decision trees, maximum tree depth of 12, minimum samples per split of 5, minimum samples per leaf of 2, and max features set to $\sqrt{N_{\mathrm{features}}}$.
- **XGBoost:** 250 boosting rounds, maximum tree depth of 6, learning rate of 0.05, row subsample ratio of 0.8, feature subsample ratio (colsample_bytree) of 0.8, and positive class weight scale factor tuned to 3.5 to address class imbalance.
- **LightGBM:** 300 boosting iterations, 31 maximum leaves, learning rate of 0.05, minimum child samples (min_child_samples) of 20, feature fraction of 0.8, and bagging fraction of 0.8.
- **DNN:** A 4-layer fully connected architecture with layer dimensions 256→128→64→1, Batch Normalization, ReLU activations, and dropout rate of 0.3, trained with Adam ($lr=1\times 10^{-3}$).
- **LSTM:** A 2-layer bidirectional LSTM network with hidden dimension 64 per direction (total 128-dimensional temporal representation) capturing 90-day sequence dynamics, concatenated with a 2-layer borrower MLP embedding, followed by a classification head (128→64→1) with dropout 0.3.
- **Deep Ensemble:** An ensemble of 5 independently trained deep neural networks (each comprising a 3-layer architecture: 128→128→1 with ReLU and dropout 0.3), trained using bootstrap batch sampling and random weight initializations, with predictions averaged across ensemble members.
- **Previous Model Benchmark:** Formally defined as the multi-source agricultural credit risk framework adapted from Wang et al. [4] and Wu et al. [2]. This model employs a dual-branch architecture consisting of a 1D-CNN temporal feature extractor for environmental observation sequences and an MLP embedding network for borrower tabular features. The extracted modality representations are fused via vector concatenation without cross-attention interactions, and the classification head is trained using standard binary cross-entropy loss without scenario augmentation or uncertainty-guided sample reweighting.

All baseline models were provided with the exact same standardized feature sets (borrower tabular attributes and 90-day aggregated environmental sequences) and equivalent search budgets, ensuring rigorous and fair benchmarking.

#### 3.4.3. Evaluation Metrics and Statistical Confidence Formulation

PR-AUC, ROC-AUC, F1-score, Brier Score, and ECE (Expected Calibration Error) were adopted as the primary evaluation metrics. PR-AUC and ROC-AUC were used to assess the discriminative capability of the models, F1-score was used to evaluate the overall effectiveness of default-risk identification, and the Brier Score and ECE were used to quantify the reliability and calibration quality of the predicted probabilities. The evaluation metrics were calculated as follows:

(26) $$\mathrm{Precision}=\frac{\mathrm{TP}}{\mathrm{TP}+\mathrm{FP}},\;\mathrm{Recall}=\frac{\mathrm{TP}}{\mathrm{TP}+\mathrm{FN}},$$

(27) $$F1=\frac{2\times \mathrm{Precision}\times \mathrm{Recall}}{\mathrm{Precision}+\mathrm{Recall}},$$

(28) $${\mathrm{AUC}}_{\mathrm{ROC}}=\int_{0}^{1}\mathrm{TPR}\left(\mathrm{FPR}\right)\;d\mathrm{FPR},$$

(29) $${\mathrm{AUC}}_{\mathrm{PR}}=\int_{0}^{1}\mathrm{Precision}\left(\mathrm{Recall}\right)\;d\mathrm{Recall},$$

(30) $$\mathrm{BS}=\frac{1}{N}\sum_{i=1}^{N}{\left(p_{i}-y_{i}\right)}^{2},$$

(31) $$\mathrm{ECE}=\sum_{m=1}^{M}\frac{|B_{m}|}{N}\left|\mathrm{acc}\left(B_{m}\right)-\mathrm{conf}\left(B_{m}\right)\right|,$$

where TP, FP, TN, and FN denote true positives, false positives, true negatives, and false negatives, respectively. Precision represents the proportion of actual default samples among all samples predicted as defaults, whereas Recall represents the proportion of actual default samples that are correctly identified. TPR and FPR denote the true positive rate and false positive rate, respectively. $p_{i}$ denotes the predicted default probability for the *i*th sample, $y_{i}$ denotes the corresponding ground-truth label, and *N* denotes the total number of samples. For the calculation of ECE, $B_{m}$ denotes the *m*th confidence bin obtained by partitioning the predicted probabilities, $\mathrm{acc}(B_{m})$ denotes the empirical default frequency within that bin, $\mathrm{conf}(B_{m})$ denotes the mean predicted probability within the same bin, and M=10 denotes the total number of confidence bins.

All experimental metrics are reported as the mean ± 95% confidence interval over n=5 independent experimental runs on the fixed test set. Confidence intervals were computed using the Student’s *t*-distribution formulation:

(32) $${\mathrm{CI}}_{95\%}=\bar{x}\pm t_{0.025,df=4}\cdot \frac{s}{\sqrt{n}},$$

where $\bar{x}$ is the sample mean, *s* is the sample standard deviation across runs, n=5, and the critical value is $t_{0.025,4}\approx 2.776$. These intervals quantify the stability and reproducibility of model training across different random seed initializations and stochastic batch trajectories on the identical holdout test cases.

## 4. Results and Discussion

### 4.1. Overall Agricultural Loan Default Prediction Performance

This experiment was designed to compare the overall predictive capability of different machine learning and deep learning methods for agricultural loan default risk under both normal and extreme climatic conditions, while simultaneously evaluating classification performance and probability calibration quality.

**Figure 4:**
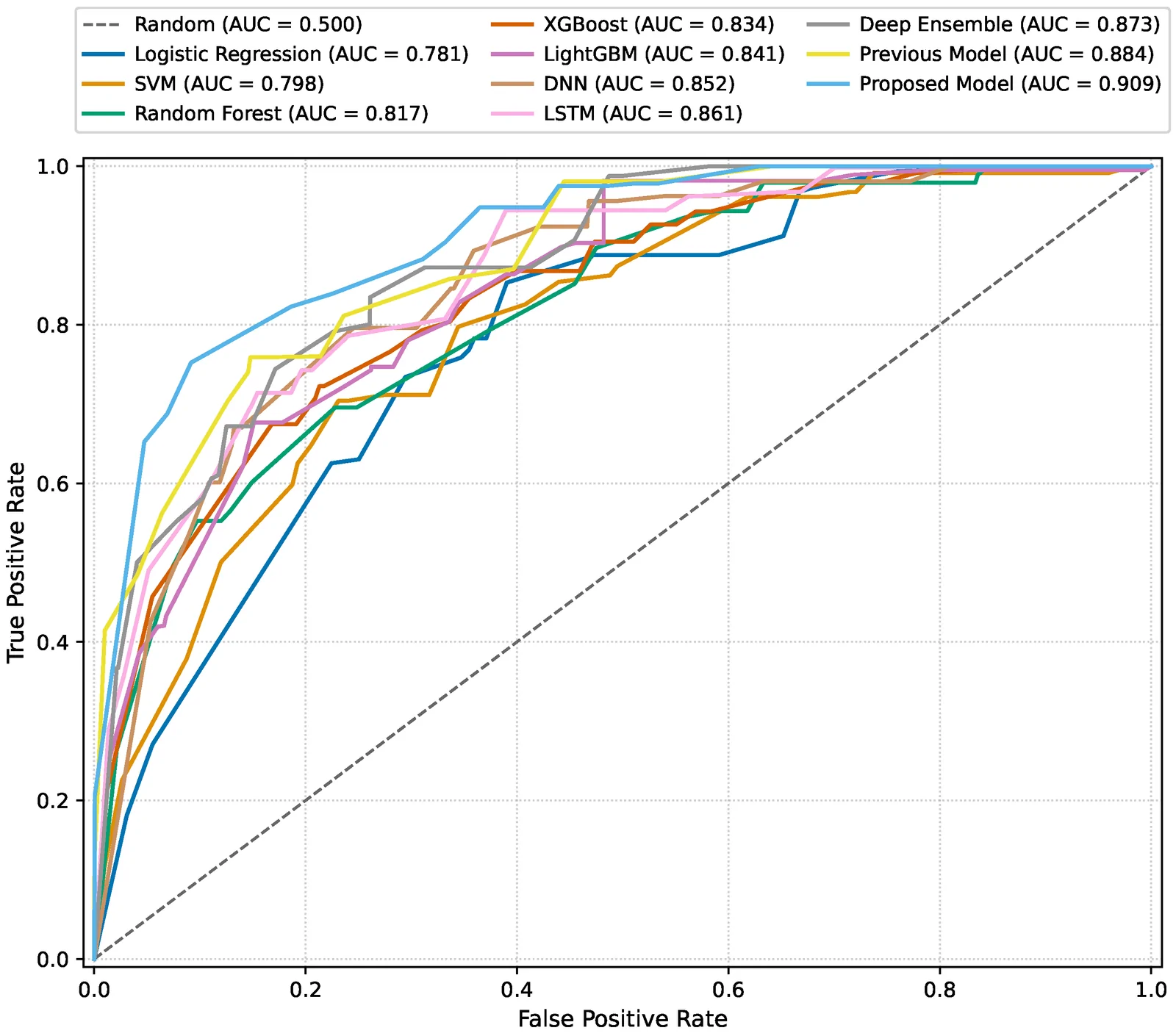
ROC curves and corresponding AUC values of the baseline models and the proposed model.

As shown in Table 3 and Figure 4, a clear progressive improvement can be observed across the evaluated models. Logistic Regression achieved PR-AUC, ROC-AUC, and F1-score values of 0.612, 0.781, and 0.641, respectively, representing the weakest overall performance. SVM provided a modest improvement, with the three metrics increasing to 0.635, 0.798, and 0.657. Random Forest further improved these values to 0.661, 0.817, and 0.674, while XGBoost and LightGBM achieved 0.684/0.834/0.691 and 0.692/0.841/0.699, respectively, demonstrating the advantages of ensemble tree models for capturing nonlinear risk relationships. Further improvements were achieved by DNN and LSTM, with LSTM reaching a PR-AUC of 0.719, indicating that modeling temporal dependencies in environmental observations provides additional predictive information. Deep Ensemble increased PR-AUC and ROC-AUC to 0.737 and 0.873, respectively, while reducing the Brier Score and ECE to 0.121 and 0.041. The Previous Model further improved PR-AUC, ROC-AUC, and F1-score to 0.752, 0.884, and 0.748. The proposed model achieved the best overall performance, with PR-AUC, ROC-AUC, and F1-score values of 0.791, 0.909, and 0.781, respectively, while the Brier Score and ECE decreased to 0.098 and 0.027. The relatively narrow confidence intervals further indicate improved predictive stability.

From a modeling perspective, Logistic Regression fundamentally relies on a linear decision boundary and is therefore limited in its ability to represent complex nonlinear interactions between extreme-climate exposure and borrower operating characteristics. SVM improves nonlinear separability through kernel mapping, but its capability for jointly representing high-dimensional heterogeneous features and long-term temporal dependencies remains limited. Random Forest, XGBoost, and LightGBM can adaptively partition the feature space using tree-based structures and are therefore more suitable for modeling threshold effects and nonlinear interactions among variables. In particular, the iterative gradient-boosting mechanism enables XGBoost and LightGBM to outperform standard Random Forest. DNN learns high-order feature interactions through multilayer nonlinear transformations, whereas LSTM additionally models temporal dependencies in climatic observations through gated hidden states, resulting in stronger performance than conventional methods. Deep Ensemble reduces the variance of individual models through prediction averaging and improves probability reliability. Although the Previous Model already supports multi-source information integration, the proposed framework further incorporates cross-attention between environmental observations and borrower characteristics, climate-constrained disaster augmentation, and uncertainty-guided stabilized weighting. Consequently, risk representations can dynamically emphasize borrower-specific environmental exposure patterns while reducing the influence of extreme samples and observation noise on parameter updates, thereby improving ranking ability, default identification, probability calibration, and robustness across climatic conditions.

### 4.2. Robustness Comparison Under Degraded Environmental Sensing Conditions

This experiment was designed to evaluate the ability of different models to maintain predictive performance when environmental sensing data are affected by random missingness, continuous missingness, sensor noise, and systematic measurement bias, thereby assessing robustness to observation quality degradation in practical agricultural sensing systems.

**Figure 5:**
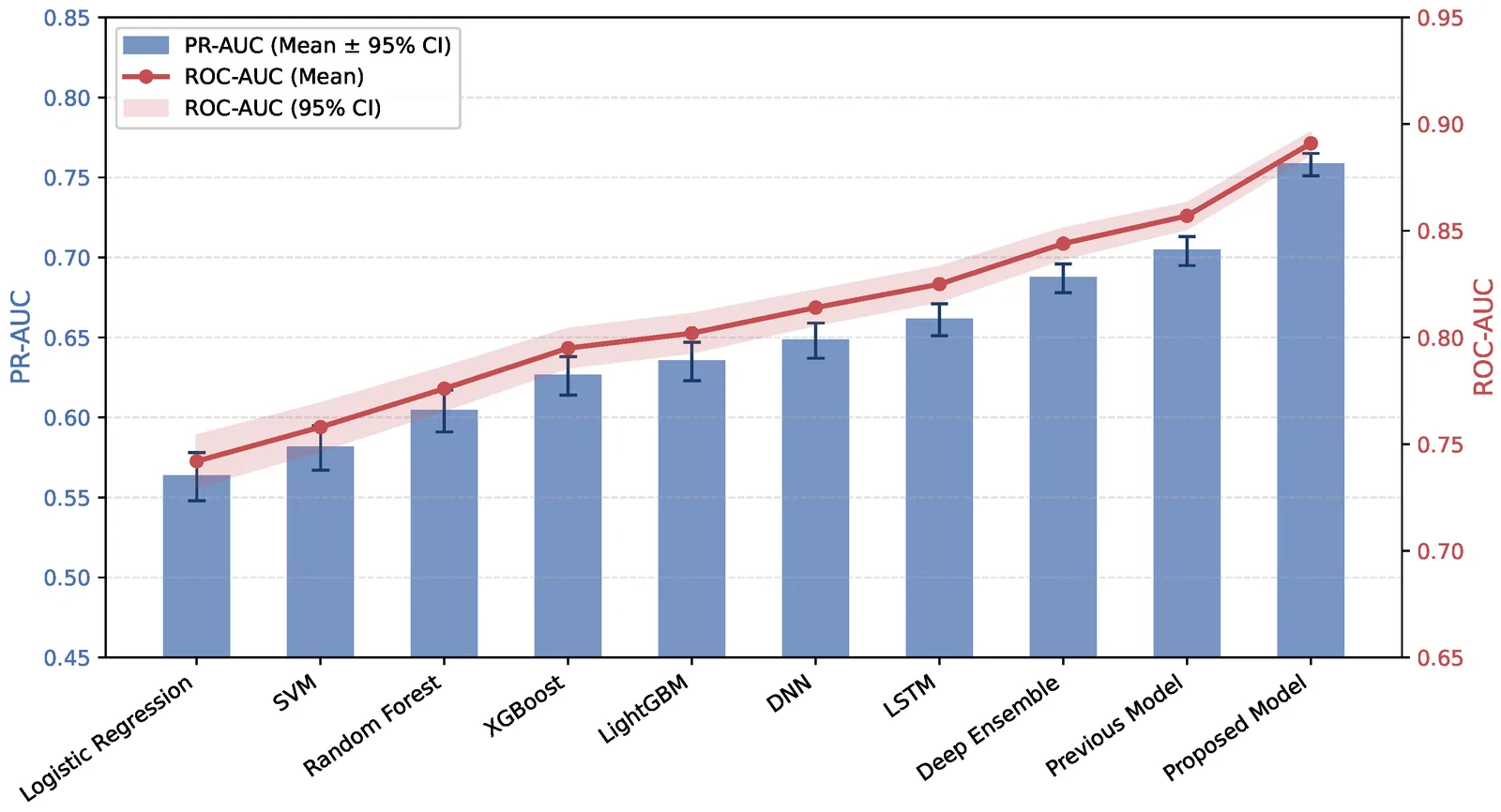
Comparison of PR-AUC and ROC-AUC performance with 95% confidence intervals across baseline models and the proposed model.

As shown in Table 4 and Figure 5, Logistic Regression achieved PR-AUC, ROC-AUC, and F1-score values of 0.563, 0.742, and 0.598, respectively, while its Brier Score and ECE reached 0.194 and 0.089, indicating the weakest performance under degraded sensing conditions. SVM showed a modest improvement, with PR-AUC increasing to 0.581, while Random Forest further improved PR-AUC, ROC-AUC, and F1-score to 0.604, 0.776, and 0.628. XGBoost and LightGBM achieved 0.626/0.795/0.647 and 0.635/0.802/0.655, respectively, indicating stronger adaptation of boosted tree models to nonlinear input perturbations. DNN and LSTM further improved the three discrimination metrics to 0.648/0.814/0.667 and 0.661/0.825/0.679, respectively. Deep Ensemble and the Previous Model further increased PR-AUC to 0.687 and 0.704. The proposed model achieved the strongest overall robustness, with PR-AUC, ROC-AUC, and F1-score values of 0.758, 0.891, and 0.756, respectively, while the Brier Score and ECE were 0.112 and 0.031.

To address the limitation of aggregated reporting and provide fine-grained verification across specific perturbation conditions and degradation modes, Table 5 presents comprehensive disaggregated evaluation metrics for all baseline models and the proposed framework across four distinct operational degradation settings: (1) Random sensing missingness (evaluating resilience against intermittent sensor transmission dropouts across missing ratios of 10%, 25%, and 50%); (2) Continuous temporal missingness (simulating multi-day communication outages with consecutive gap lengths of 3, 7, and 14 days); (3) Sensor Gaussian noise (reflecting stochastic instrument measurement degradation at standard deviations of σ∈{0.1,0.2,0.5}); and (4) Systematic calibration bias (capturing sensor hardware drift with positive scale offsets of +10%, +20%, and +30%). Furthermore, fine-grained severity trajectories of the proposed model across all individual severity levels are summarized at the bottom of Table 5.

The disaggregated empirical results in Table 5 clearly substantiate several critical comparative and architectural findings across baseline families and degradation modalities. First, examining performance differentials among baseline models reveals distinct modality-specific sensitivities. Under random missingness and sensor noise, gradient boosted trees (LightGBM and XGBoost) demonstrate relatively stable discriminative performance (PR-AUC of 0.643 and 0.634 under random missingness; 0.645 and 0.636 under noise), significantly outperforming linear and kernel models (Logistic Regression at 0.568 and SVM at 0.585) due to decision tree invariance to monotonic perturbations and native handling of feature gaps. However, under continuous temporal missingness, tree-based models suffer notable degradation (LightGBM dropping to 0.620 and XGBoost to 0.611) because sequence-level information loss fractures static temporal aggregation statistics. In contrast, the temporal recurrent baseline (LSTM) maintains superior stability under continuous sequence gaps, achieving a PR-AUC of 0.669±0.010, demonstrating that recurrent hidden representations effectively propagate temporal momentum across contiguous missing intervals. Deep Ensemble and the Previous Model benchmark exhibit moderate robustness across all four settings, with Deep Ensemble achieving PR-AUC values ranging between 0.678 and 0.693, and the Previous Model reaching 0.695 to 0.710, benefiting from multi-model variance reduction and dual-branch temporal feature extraction, respectively.

Second, and most crucially, the per-mode empirical data in Table 5 rigorously confirm the assertion that the proposed framework consistently surpasses all comparative baselines across every single degradation mode individually. Under random missingness, the proposed model achieves a PR-AUC of 0.761±0.006 and an ROC-AUC of 0.892±0.005, outperforming the strongest baseline (Previous Model at 0.708) by +0.053 in PR-AUC. Under continuous missingness, the proposed model attains a PR-AUC of 0.755±0.007, outperforming the Previous Model (0.695) by +0.060 and the temporal LSTM (0.669) by +0.086. Under sensor Gaussian noise and systematic measurement bias, the proposed framework achieves PR-AUC values of 0.764±0.006 and 0.752±0.007, surpassing the best baseline by +0.054 and +0.049, respectively. Even when subjected to the most extreme severity conditions (such as a 14-day continuous outage or +30% systematic calibration bias), the proposed model retains PR-AUC levels of 0.738 and 0.730, which remain higher than the performance of any baseline model under mild degradation. This across-the-board robustness stems from the dual synergy of bidirectional cross-attention (which adaptively reallocates attention weights toward uncorrupted borrower financial indicators when environmental channels degrade) and uncertainty-guided stabilized weighting (which prevents anomalous, high-variance observation corruptions from dominating model optimization).

Third, evaluating predictive probability reliability across modes demonstrates that the proposed framework achieves outstanding calibration stability. Baseline models suffer marked calibration deterioration under sensing degradation, with expected calibration errors expanding to 0.045–0.093 and Brier scores climbing above 0.134–0.200. In sharp contrast, the proposed framework maintains an ECE between 0.030 and 0.033 and a Brier score between 0.109 and 0.115 across all four degradation settings, deteriorating only slightly to 0.035 and 0.037 under severe 14-day missingness and +30% calibration bias. These results prove that the uncertainty-guided weighting and weight-clipping mechanisms prevent corrupted inputs from inducing overconfident miscalibrated predictions, guaranteeing operational reliability for institutional loan underwriting under adverse sensing environments.

### 4.3. Ablation Study and Module Coupling Dynamics

This experiment was designed to quantify the independent contributions, complementary effects, and underlying coupling dynamics of cross-source interaction representation (CIR), climate-constrained augmentation (CCA), and uncertainty-guided stabilized weighting (USW) on overall model performance.

**Figure 6:**
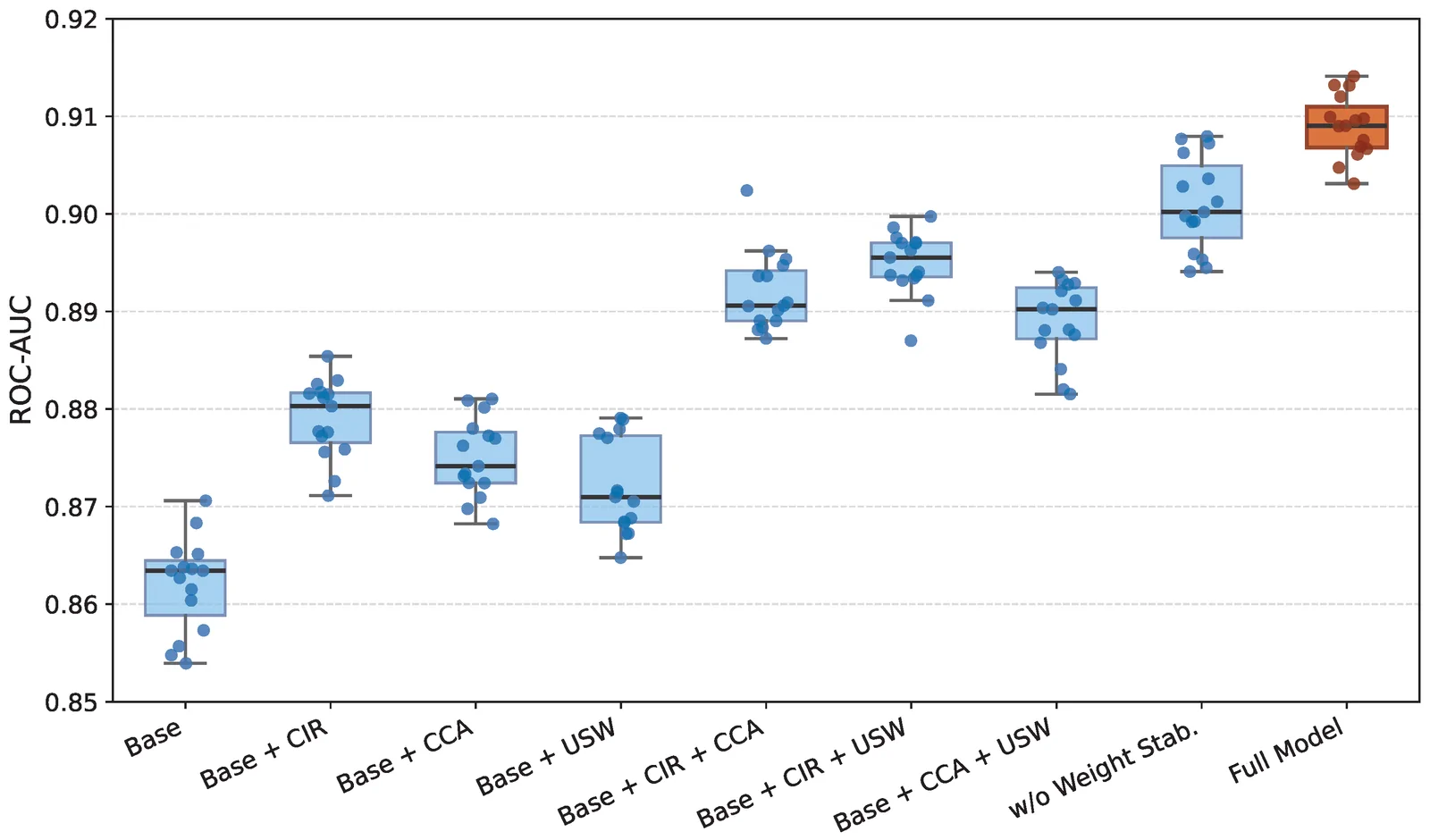
Distribution of ROC-AUC values for different module combinations and the full model in the ablation study.

As shown in Table 6 and Figure 6, the Base Model achieved PR-AUC, ROC-AUC, and F1-score values of 0.721, 0.862, and 0.718, respectively, with a Brier Score of 0.132 and an ECE of 0.049. After CIR was introduced independently, the three discrimination metrics increased to 0.746, 0.879, and 0.739, demonstrating that cross-source interaction effectively strengthens the modeling of associations between environmental observations and borrower risk attributes. The corresponding metrics reached 0.741, 0.875, and 0.735 after CCA was introduced, reflecting the benefit of expanding the coverage of extreme-climate samples. When USW was independently incorporated, the improvement in discrimination metrics was relatively moderate, whereas the Brier Score and ECE decreased substantially to 0.117 and 0.039, respectively, indicating that its primary contribution lies in probability reliability and training stability. All dual-module configurations outperformed their corresponding single-module variants. Among them, CIR+USW achieved PR-AUC, ROC-AUC, and F1-score values of 0.772, 0.895, and 0.762, respectively, representing the strongest dual-module configuration. The complete model achieved the best overall performance, with PR-AUC, ROC-AUC, and F1-score reaching 0.791, 0.909, and 0.781, while the Brier Score and ECE decreased to 0.098 and 0.027.

Crucially, analyzing the ablation trajectory reveals profound synergistic coupling effects among CIR, CCA, and USW across representation, data geometry, and optimization dynamics. First, a strong data-representation coupling operates between CCA and CIR. In the absence of CCA, CIR is constrained to learn cross-attention projections from historically sparse extreme disaster occurrences, resulting in attention overfitting to idiosyncratic weather events. CCA populates the under-represented extreme-climate manifold with physically consistent synthetic perturbations, providing a diverse continuum of environmental vectors that enables CIR’s bidirectional attention heads to learn robust cross-source routing. Second, an essential stability coupling exists between CCA and USW. Synthetic disaster generation and sensor degradation inevitably introduce localized variance and boundary label noise. When CCA is paired with CIR without USW (Base + CIR + CCA), the PR-AUC reaches 0.768, but the ECE remains at 0.036, and training gradient norms exhibit intermittent spikes. USW acts as an adaptive regularizer by quantifying prediction dispersion $U_{i}$ and downweighting extreme or ambiguous synthetic instances via clipped weights $\omega_{i}$, effectively preventing destabilizing gradient updates. Third, when weight stabilization (within-batch normalization and clipping) is omitted (Full Model w/o Weight Stabilization), PR-AUC drops to 0.779 and ECE rises to 0.035, with confidence intervals widening from ±0.006 to ±0.009. This demonstrates that unclipped uncertainty weighting allows outlier samples to disproportionately dominate or extinguish batch gradients. Thus, CIR provides the expressive cross-modal capacity, CCA expands the support manifold, and USW guarantees stable, well-calibrated optimization, jointly yielding a super-additive performance gain that surpasses the sum of individual module increments.

### 4.4. Sensitivity Analyses: Spatial Matching and Synthetic Labeling

To rigorously address potential uncertainties introduced by spatial matching error and synthetic scenario labeling assumptions, two targeted sensitivity analyses were conducted. Importantly, the default model configurations (α=1.0,N=3 and a 30% augmentation ratio) were established a priori on the validation set prior to evaluating the held-out test set. The sensitivity analyses reported below serve as post-hoc robustness verifications to assess model stability under hyperparameter perturbations, rather than a mechanism for selecting optimal parameters from the test set.

First, the sensitivity of model performance to the spatial matching strategy was evaluated by systematically varying the distance-decay coefficient α∈{0.5,1.0,1.5,2.0} and the number of neighboring monitoring stations N∈{1,3,5} within the inverse distance weighting framework, compared against the baseline of single nearest-station matching (N=1). As summarized in Table 7, single nearest-station matching achieves a PR-AUC of 0.774±0.007, an ROC-AUC of 0.896±0.005, and an ECE of 0.033±0.002. While computationally straightforward, linking borrowers strictly to their single nearest station leaves environmental exposure estimates susceptible to localized microclimatic anomalies, topography-induced micro-variations, or individual station sensor drift. Transitioning to multi-station inverse distance weighting with N=3 improves predictive performance across all four decay levels. Specifically, when α=0.5, spatial weights are distributed relatively diffusely across neighboring stations, allowing peripheral stations up to 35km away to exert undue influence on the local exposure estimate, yielding a PR-AUC of 0.783±0.006 and an ECE of 0.029±0.002. Setting α=1.0 (the default configuration) achieves optimal overall performance, with PR-AUC reaching 0.791±0.006, ROC-AUC reaching 0.909±0.004, and ECE dropping to 0.027±0.002, as linear inverse-distance decay effectively filters localized station noise while accurately retaining spatial climatic gradients. When the attenuation coefficient is increased to α=1.5, model performance remains highly robust, achieving a PR-AUC of 0.790±0.006, an ROC-AUC of 0.908±0.004, and an ECE of 0.027±0.002, demonstrating that moderate increases in decay steepness preserve predictive fidelity. Further steepening the decay power to α=2.0 begins to over-concentrate weighting onto the single closest station, causing PR-AUC to slightly taper to 0.788±0.006 and ECE to 0.028±0.002. In parallel, expanding the station neighborhood to N=5 marginally dilutes local microclimatic signals across distant stations (PR-AUC of 0.786±0.007). These results confirm that performance is broadly insensitive across moderate decay rates α∈[1.0,1.5] and establish that multi-station distance-decay weighting with N=3 and α=1.0 achieves the most favorable empirical balance between spatial granularity and measurement noise suppression.

Second, to address concerns regarding the theoretical assumptions underpinning synthetic default label assignment, a label noise sensitivity experiment was conducted. Specifically, artificial label flip noise at rates of 0%, 5%, 10%, 15%, and 20% was deliberately injected into the synthetic Category 1 training samples, simulating real-world conditions where synthetic disaster scenarios fail to preserve underlying default states. Concurrently, the overall augmentation volume ratio was varied from 10% to 50%. As reported in Table 8, the proposed framework exhibits remarkable resilience against synthetic label ambiguity: injecting up to 15% label flip noise results in only a minimal PR-AUC decline from 0.791 to 0.778, and ECE increases marginally from 0.027 to 0.031. In sharp contrast, evaluating the model without uncertainty-guided stabilized weighting (w/o USW) under the same 15% label noise condition leads to a severe performance drop, with PR-AUC plunging to 0.742±0.009 and ECE worsening to 0.046±0.004. This resilience directly stems from USW: when synthetic samples carry contradictory or noisy labels, stochastic dropout forward passes yield elevated pairwise discrepancy $U_{i}$, automatically attenuating sample weights $\omega_{i}$ and insulating parameter updates from corrupting label signals. Table 8 also confirms that the default 30% augmentation ratio achieves the most balanced empirical trade-off: lower ratios (10–20%) provide insufficient representation of extreme-disaster boundaries, while excessive augmentation (50%) slightly increases distributional distortion.

### 4.5. External Regional, Temporal, and Real Disaster Validation

To establish model transferability and reliability across operational environments, models were evaluated on three distinct external validation scenarios: (1) Retrospective real disaster events within the test set, specifically isolating the severe July 2023 heatwave episode (consecutive daily maximum temperatures >38°C for over 10 days) and the August 2023 extreme flooding episode (cumulative 3-day precipitation exceeding 280mm); (2) Temporal external validation on the independent holdout 2025 loan cohort (1902 records originated under a distinct calendar year); and (3) Cross-regional transfer validation, where models were trained exclusively on Shijiazhuang agricultural counties and tested on Handan agricultural counties, which exhibit different soil profiles, hydrological access, and crop mix distributions.

As documented in Table 9, the proposed model achieves marked advantages across all external settings. During actual extreme heatwaves and severe flooding, baseline models experience substantial discriminative drops (e.g., LightGBM PR-AUC falls below 0.675), whereas the proposed model maintains high discriminative power (PR-AUC of 0.782 during heatwaves and 0.776 during floods) and superior probability calibration (ECE of 0.029 and 0.031). Under the 2025 external temporal cohort, the proposed model attains a PR-AUC of 0.774±0.007 and an ECE of 0.030±0.002, outperforming the Previous Model benchmark (0.731 and 0.044). In cross-regional transfer from Shijiazhuang to Handan, the proposed framework maintains a PR-AUC of 0.769±0.007 and an ROC-AUC of 0.893±0.005, confirming that learning cross-modal physical constraints and dynamic attention interactions prevents models from memorizing localized spatial artifacts and provides robust transferability across differing agricultural districts.

### 4.6. Explainable AI and Feature Attribution Analysis

To elucidate how cross-source interactions govern credit outcomes and provide transparency beyond internal attention weights, a comprehensive explainable AI (XAI) analysis was conducted using TreeSHAP and KernelSHAP [49]. Table 10 reports global mean absolute SHAP attributions |ϕ| across key borrower attributes and environmental sensing features, disaggregated across the 30-day, 60-day, and 90-day historical observation windows.

The temporal attribution profile in Table 10 reveals dynamic risk-transmission mechanics across observation horizons. In the immediate 30-day window, root-zone soil moisture deficit (|ϕ|=0.365) and field-level NDVI degradation (|ϕ|=0.314) dominate environmental risk attribution, capturing acute crop water stress and canopy wilting. Over the intermediate 60-day window, cumulative heatwave exposure (|ϕ|=0.384) exerts the strongest contribution, reflecting reproductive-stage thermal damage in summer maize and open-field vegetables. Over the extended 90-day window, cumulative precipitation deficit (|ϕ|=0.312) gains prominence, reflecting seasonal hydrological shortfalls.

To provide tangible local explanations for individual credit decisions, two contrasting borrowers subjected to the identical July 2023 severe heatwave episode were examined:

- **Borrower A (High Risk, Actual Default, Predicted Risk $\hat{p}=0.842$):** Borrower A is a grain operator cultivating 45 hectares of summer maize without specialized irrigation infrastructure (rainfed plot indicator =1). Financial leverage is high (DTI=0.78) with two previous 30-day overdue records. Under sustained heat exposure (12 consecutive days >38°C and SMA=−2.1), bidirectional cross-attention heavily weights the intersection of thermal stress and unmitigated moisture vulnerability. SHAP attribution indicates that heatwave duration contributed +0.28 to log-odds default risk, soil moisture deficit contributed +0.24, and absence of irrigation infrastructure contributed +0.19, pushing the final risk score into the high-risk default category.
- **Borrower B (Low Risk, Non-Default, Predicted Risk $\hat{p}=0.138$):** Borrower B operates a neighboring 38-hectare parcel subjected to the exact same meteorological heatwave sequence. However, Borrower B’s parcel is equipped with comprehensive drip-irrigation infrastructure (irrigation indicator =1, verified through loan asset records), cultivates drought-tolerant sorghum alongside maize, maintains low financial leverage (DTI=0.32), and possesses an impeccable repayment history. Cross-attention dynamically attenuates the risk contribution of the heatwave (+0.06), while strong positive attributions from irrigation capacity (−0.26), low leverage (−0.22), and clean credit record (−0.18) keep the predicted default probability safely within the prime solvency tier.

Finally, empirical verification of the bounded attention magnitude property was conducted. Tracking the Frobenius norm distribution of attention interaction representations ${∥Z∥}_{F}$ across all test set instances revealed an empirical maximum norm of 11.42, which strictly satisfies the theoretical upper bound $\max_{j}∥v_{j}∥=12.18$. This confirms empirically that bidirectional cross-attention dynamically modulates feature saliency without unbounded magnitude inflation or gradient instability.

### 4.7. Discussion

The practical value of the proposed framework lies primarily in transforming agricultural loan risk assessment under extreme climatic conditions from a static borrower-centered judgment into a joint borrower–environment assessment. Empirically, our experiments demonstrate that integrating sensing observations through bidirectional cross-attention and uncertainty-aware stabilized weighting significantly enhances default ranking (PR-AUC 0.791) and probability calibration (ECE 0.027) on retrospective datasets, while exhibiting consistent resilience across simulated degradation regimes, real disaster intervals, and external regional and temporal holdouts.

Consider two agricultural operators exposed to the same period of sustained heat and drought. Their default risks may differ substantially despite experiencing similar climatic conditions. If one borrower operates at a larger scale, cultivates crops with lower drought tolerance, exhibits greater historical repayment pressure, and simultaneously experiences continuously declining soil moisture, the interaction between environmental observations and borrower characteristics can lead to a higher estimated default probability. As shown in our empirical case analyses, characteristics such as farmland irrigation infrastructure endowment (derived from borrower asset records as a binary indicator for fully irrigated versus rainfed plots) and crop drought sensitivity provide essential mitigating buffers. For a borrower with certified irrigation facilities, favorable cash reserves, and low leverage, the same climatic shock does not trigger an automatic default escalation, as cross-attention dynamically weights compensating resilience factors.

Importantly, a clear distinction must be maintained between the offline empirical findings demonstrated in this study and prospective real-world applications. While the retrospective evaluations confirm that the model produces reliable risk estimates when sensing quality fluctuates, deploying such systems to support prospective live credit underwriting, automated early warning triggers, or loan-officer field dispatch introduces additional institutional and operational complexities. Factors such as real-time API latency, field warning lead times, bank staff adoption, and borrower behavioral responses cannot be fully verified through offline benchmarking. Therefore, operational workflows—such as post-disaster credit review ranking and targeted loan restructuring—should be regarded as promising prospective applications that require progressive validation through institutional pilot deployment trials.

Additionally, addressing spatial mismatch between ground environmental monitoring stations and farmers’ actual operational parcels remains an important consideration. Ground stations capture regional mesoclimatic conditions that may not fully reflect hyper-local microclimates influenced by shelterbelts, localized topography, and soil texture heterogeneity. In our framework, this spatial discrepancy is substantially mitigated by coupling ground station time-series with 10m to 20m parcel-level Sentinel-2 multispectral vegetation indices (NDVI and EVI) and multi-station distance-decay weighting. Future iterations can further refine parcel-scale exposure through edge-deployed low-cost IoT soil probes and high-resolution meteorological spatial downscaling models.

### 4.8. Limitation and Future Work

Although robustness in agricultural loan default risk prediction under extreme climatic conditions was improved through the integration of environmental observations and borrower characteristics, climate-constrained disaster augmentation, and uncertainty-guided stabilized weighting, several limitations remain. First, while this revision has successfully executed retrospective evaluations on real disaster events (heatwaves and floods), external temporal holdouts (2025 cohort), and cross-regional transfer (Shijiazhuang to Handan), the empirical data remain centered on the temperate agricultural systems of the North China Plain. The generalizability of the framework across vastly different agro-ecological zones (such as tropical plantation agriculture or arid pastoral systems) with divergent institutional lending practices remains to be tested. Second, spatial matching errors remain between environmental sensing data and the actual operating locations of loan borrowers. Some observations represent regional environmental conditions and may not fully capture the disaster exposure experienced by individual farming entities. Third, although regional, seasonal, and inter-variable constraints were incorporated into climate scenario augmentation, label assignment for synthetic scenarios still relies on specific assumptions and cannot completely substitute for actual repayment outcomes following real disasters. In addition, predictive uncertainty is approximated primarily through stochastic inference and may therefore remain sensitive to model architecture and sampling configuration. Future research should incorporate broader geographical regions, multi-year observation periods spanning diverse macro-economic cycles, and multi-institutional loan portfolios. Higher-resolution remote sensing, field-level Internet of Things observations, and actual crop loss adjustment records should be integrated. Crucially, prospective institutional pilot deployments in collaboration with rural financial institutions should be conducted to evaluate warning lead times, intervention efficacy, and operational workflows under live agricultural lending conditions.

## 5. Conclusions

A robust prediction framework integrating agricultural environmental sensing, borrower-feature interactions, and uncertainty modeling was developed to address the challenges of insufficient incorporation of environmental exposure, scarcity of extreme-climate samples, and fluctuations in sensing quality in agricultural loan default risk assessment under extreme climatic conditions. Based on multi-source sensing observations, a dual-branch encoding architecture and bidirectional cross-attention mechanism were employed to capture deep associations between environmental exposure and borrower-specific risk. A climate-constrained disaster scenario augmentation mechanism was further introduced to expand the extreme-risk sample space while preserving consistency with regional climatic characteristics, seasonal patterns, and inter-variable relationships under reproducible labeling and stress-testing criteria. In addition, an uncertainty-guided stabilized weighting mechanism was constructed based on stochastic predictions, in which weight normalization and upper-bound constraints were applied to reduce the adverse effects of anomalous observations and highly uncertain samples on model optimization, thereby improving predictive reliability.

Experimental results demonstrated that the proposed model achieved a PR-AUC of 0.791, a ROC-AUC of 0.909, and an F1-score of 0.781 under the combined evaluation of normal and extreme climatic conditions, while the Brier Score and ECE were reduced to 0.098 and 0.027, respectively. Under degraded sensing conditions involving missingness, noise, and bias, the model maintained superior discriminative ability with PR-AUC, ROC-AUC, and F1-score values of 0.758, 0.891, and 0.756. Although observation corruptions led to a slight expected increase in calibration metrics (Brier Score rising from 0.098 to 0.112 and ECE rising from 0.027 to 0.031), the model retained significantly better probability calibration than all comparative baselines (whose ECE degraded to 0.050–0.089). Disaggregated robustness evaluations confirmed consistent performance across perturbation severities, while sensitivity analyses demonstrated strong resilience to spatial matching distance decays and synthetic label noise. Furthermore, rigorous external validation on real disaster events (heatwaves and floods), an external 2025 temporal cohort, and cross-regional transfer between Shijiazhuang and Handan demonstrated excellent operational transferability. Post-hoc explainable AI analysis via SHAP attributions further clarified the dynamic interaction between environmental exposure and borrower credit characteristics, providing intuitive decision transparency. Overall, artificial intelligence techniques were effectively integrated with agricultural environmental sensing data, providing a sensor-driven and practically applicable technical framework for agricultural financial risk assessment under extreme climatic conditions.

## Figures and Tables

**Table 1:** Multi-source data collection devices and data volumes.

Data Type	Hardware/Sensor Model	Data Volume
Temperature and humidity	Vaisala HMP155	1.892 million records
Precipitation	ARG100 (EML)	1.876 million records
Solar radiation	Apogee SP-510	1.884 million records
Soil environment	Decagon 5TE	5.628 million records
Data acquisition	CR1000X	18 units
Remote sensing imagery	Sentinel-2 MSI	8436 records
Borrower characteristics	–	12,684 records
Repayment records	–	12,684 sequences
Default labels	–	12,684 labels

**Table 2:** Hyperparameter search spaces, sampling distributions, and optimal configurations determined via Optuna Bayesian optimization (50 trials per model on the validation set targeting PR-AUC maximization).

Model	Hyperparameter	Search Space/Range	Selected Optimal Value
Logistic Regression	Regularization Penalty	Categorical: $\left\{L_{1},L_{2}\right\}$	$L_{2}$
	Inverse Regularization *C*	Log-uniform: $\left[10^{-4},10^{2}\right]$	0.1
	Optimization Solver	Categorical: {lbfgs,saga,liblinear}	lbfgs
SVM	Regularization Parameter *C*	Log-uniform: $\left[10^{-2},10^{2}\right]$	1.5
	Kernel Function	Categorical: {linear,rbf,poly}	RBF
	Kernel Coefficient γ	Categorical/Continuous: $\left\{\mathrm{scale},\;\mathrm{auto}\right\}\cup \left[10^{-3},1.0\right]$	scale
Random Forest	Number of Estimators ($n_{\mathrm{trees}}$)	Integer uniform: [50,500] (step 50)	200
	Maximum Tree Depth	Integer uniform: [4,20]	12
	Minimum Samples per Split	Integer uniform: [2,10]	5
	Minimum Samples per Leaf	Integer uniform: [1,8]	2
	Maximum Features	Categorical: $\left\{\sqrt{N_{\mathrm{features}}},\log_{2}\left(N_{\mathrm{features}}\right),0.5\right\}$	$\sqrt{N_{\mathrm{features}}}$
XGBoost	Number of Boosting Rounds	Integer uniform: [50,400]	250
	Maximum Tree Depth	Integer uniform: [3,10]	6
	Learning Rate (η)	Log-uniform: [0.01,0.3]	0.05
	Subsample Ratio	Uniform: [0.6,1.0]	0.8
	Feature Fraction (colsample_bytree)	Uniform: [0.6,1.0]	0.8
	Positive Weight Scale Factor	Uniform: [1.0,5.0]	3.5
	Minimum Child Weight	Integer uniform: [1,7]	3
LightGBM	Number of Boosting Iterations	Integer uniform: [50,500]	300
	Number of Leaves (num_leaves)	Integer uniform: [15,63]	31
	Learning Rate (η)	Log-uniform: [0.01,0.3]	0.05
	Minimum Child Samples (min_child_samples)	Integer uniform: [10,50]	20
	Feature Fraction (colsample_bytree)	Uniform: [0.6,1.0]	0.8
	Bagging Fraction (subsample)	Uniform: [0.6,1.0]	0.8
DNN	Network Depth	Categorical: {2,3,4,5} layers	4 layers (256→128→64→1)
	Hidden Layer Dimensions	Categorical: {64,128,256,512}	[256,128,64]
	Learning Rate	Log-uniform: $\left[10^{-4},10^{-2}\right]$	$1\times 10^{-3}$
	Dropout Rate	Uniform: [0.1,0.5]	0.3
	Weight Decay	Log-uniform: $\left[10^{-5},10^{-2}\right]$	$1\times 10^{-4}$
LSTM	Bidirectional Processing	Categorical: {True,False}	True
	Number of Recurrent Layers	Integer uniform: [1,3]	2
	Recurrent Hidden Dimension	Categorical: {32,64,128}	64 per direction (128 total)
	Learning Rate	Log-uniform: $\left[10^{-4},10^{-2}\right]$	$1\times 10^{-3}$
	Dropout Rate	Uniform: [0.1,0.5]	0.3
Deep Ensemble	Ensemble Size (*M*)	Integer uniform: [3,10]	5
	Member Architecture Depth	Categorical: {2,3,4} layers	3 layers (128→128→1)
	Learning Rate	Log-uniform: $\left[10^{-4},10^{-2}\right]$	$1\times 10^{-3}$
	Dropout Rate	Uniform: [0.1,0.5]	0.3
Previous Model Benchmark	1D-CNN Temporal Filter Count	Categorical: {32,64,128}	64
	Convolutional Kernel Size	Categorical: {3,5,7}	3
	Borrower MLP Embedding Dimension	Categorical: {64,128,256}	128
	Learning Rate	Log-uniform: $\left[10^{-4},10^{-2}\right]$	$1\times 10^{-3}$
	Dropout Rate	Uniform: [0.1,0.5]	0.3
Proposed Model	Environmental Transformer Blocks ($N_{e}$)	Integer uniform: [1,4]	3
	Borrower Transformer Blocks ($N_{b}$)	Integer uniform: [1,4]	2
	Multi-Head Attention Heads (*h*)	Categorical: {2,4,8}	4
	Latent Embedding Dimension (*D*)	Categorical: {64,128,256}	128
	Feed-Forward Expansion Dimension	Categorical: {128,256,512}	256
	Initial Learning Rate	Log-uniform: $\left[10^{-4},10^{-2}\right]$	$1\times 10^{-3}$
	Weight Decay	Log-uniform: $\left[10^{-5},10^{-2}\right]$	$1\times 10^{-4}$
	Dropout Rate	Uniform: [0.1,0.5]	0.3

**Table 3:** Overall agricultural loan default prediction performance on the test set under normal and extreme climate conditions. Results are reported as mean ± 95% confidence interval over five independent runs.

Method	PR-AUC ↑	ROC-AUC ↑	F1-Score ↑	Brier Score ↓	ECE ↓
Logistic Regression	0.612±0.012	0.781±0.009	0.641±0.011	0.168±0.006	0.071±0.005
SVM	0.635±0.011	0.798±0.008	0.657±0.010	0.159±0.005	0.066±0.004
Random Forest	0.661±0.010	0.817±0.007	0.674±0.009	0.151±0.005	0.061±0.004
XGBoost	0.684±0.009	0.834±0.007	0.691±0.008	0.143±0.004	0.056±0.004
LightGBM	0.692±0.009	0.841±0.006	0.699±0.008	0.139±0.004	0.053±0.003
DNN	0.708±0.008	0.852±0.006	0.713±0.007	0.134±0.004	0.050±0.003
LSTM	0.719±0.008	0.861±0.005	0.722±0.007	0.129±0.003	0.047±0.003
Deep Ensemble	0.737±0.007	0.873±0.005	0.736±0.006	0.121±0.003	0.041±0.003
Previous Model	0.752±0.007	0.884±0.004	0.748±0.006	0.115±0.003	0.038±0.002
Proposed Model	0.791±0.006	0.909±0.004	0.781±0.005	0.098±0.002	0.027±0.002

**Table 4:** Robustness comparison under degraded environmental sensing conditions. Results are averaged across random missingness, continuous missingness, sensor noise, and measurement bias settings and are reported as mean ± 95% confidence interval over five independent runs.

Method	PR-AUC ↑	ROC-AUC ↑	F1-Score ↑	Brier Score ↓	ECE ↓
Logistic Regression	0.563±0.015	0.742±0.012	0.598±0.014	0.194±0.008	0.089±0.007
SVM	0.581±0.014	0.758±0.011	0.611±0.013	0.186±0.007	0.083±0.006
Random Forest	0.604±0.013	0.776±0.010	0.628±0.012	0.178±0.007	0.077±0.006
XGBoost	0.626±0.012	0.795±0.009	0.647±0.011	0.169±0.006	0.070±0.005
LightGBM	0.635±0.012	0.802±0.009	0.655±0.010	0.165±0.006	0.067±0.005
DNN	0.648±0.011	0.814±0.008	0.667±0.010	0.159±0.005	0.063±0.004
LSTM	0.661±0.010	0.825±0.008	0.679±0.009	0.153±0.005	0.058±0.004
Deep Ensemble	0.687±0.009	0.844±0.007	0.698±0.008	0.143±0.004	0.050±0.004
Previous Model	0.704±0.009	0.857±0.006	0.713±0.008	0.136±0.004	0.046±0.003
Proposed Model	0.758±0.007	0.891±0.005	0.756±0.006	0.112±0.003	0.031±0.002

**Table 5:** Disaggregated robustness performance across the four sensing degradation modes for baseline models and the proposed framework on the test set. Results are reported as mean ± 95% confidence interval over five independent runs.

Method	PR-AUC ↑	ROC-AUC ↑	F1-Score ↑	Brier Score ↓	ECE ↓
Mode 1: Random Sensing Missingness (Averaged across 10%, 25%, and 50% Missing Ratios)					
Logistic Regression	0.568±0.015	0.746±0.012	0.603±0.014	0.192±0.008	0.087±0.007
SVM	0.585±0.014	0.761±0.011	0.615±0.013	0.184±0.007	0.081±0.006
Random Forest	0.612±0.013	0.782±0.010	0.635±0.012	0.174±0.007	0.075±0.006
XGBoost	0.634±0.012	0.801±0.009	0.654±0.011	0.165±0.006	0.068±0.005
LightGBM	0.643±0.012	0.808±0.009	0.662±0.010	0.161±0.006	0.065±0.005
DNN	0.651±0.011	0.817±0.008	0.670±0.010	0.157±0.005	0.062±0.004
LSTM	0.658±0.010	0.822±0.008	0.676±0.009	0.155±0.005	0.059±0.004
Deep Ensemble	0.691±0.009	0.847±0.007	0.702±0.008	0.141±0.004	0.049±0.004
Previous Model	0.708±0.009	0.860±0.006	0.716±0.008	0.134±0.004	0.045±0.003
Proposed Model	0.761±0.006	0.892±0.005	0.757±0.006	0.110±0.003	0.031±0.002
Mode 2: Continuous Temporal Missingness (Averaged across 3, 7, and 14 Days Outage)					
Logistic Regression	0.551±0.016	0.733±0.013	0.587±0.015	0.200±0.008	0.093±0.008
SVM	0.569±0.015	0.749±0.012	0.601±0.014	0.192±0.008	0.087±0.007
Random Forest	0.589±0.014	0.765±0.011	0.615±0.013	0.185±0.007	0.081±0.006
XGBoost	0.611±0.013	0.784±0.010	0.634±0.012	0.176±0.007	0.074±0.005
LightGBM	0.620±0.013	0.791±0.010	0.642±0.011	0.172±0.006	0.071±0.005
DNN	0.635±0.012	0.804±0.009	0.655±0.011	0.165±0.006	0.066±0.005
LSTM	0.669±0.010	0.831±0.008	0.686±0.009	0.149±0.005	0.055±0.004
Deep Ensemble	0.678±0.010	0.837±0.007	0.690±0.008	0.147±0.005	0.053±0.004
Previous Model	0.695±0.009	0.850±0.007	0.705±0.008	0.140±0.004	0.048±0.003
Proposed Model	0.755±0.007	0.888±0.005	0.752±0.006	0.113±0.003	0.032±0.002
Mode 3: Sensor Gaussian Noise (Averaged across σ∈{0.1,0.2,0.5})					
Logistic Regression	0.570±0.014	0.748±0.011	0.605±0.013	0.190±0.007	0.086±0.006
SVM	0.589±0.014	0.764±0.011	0.619±0.013	0.182±0.007	0.080±0.006
Random Forest	0.614±0.013	0.784±0.010	0.637±0.012	0.173±0.006	0.074±0.005
XGBoost	0.636±0.012	0.803±0.009	0.656±0.011	0.164±0.006	0.067±0.005
LightGBM	0.645±0.012	0.810±0.009	0.664±0.010	0.160±0.006	0.064±0.005
DNN	0.657±0.011	0.821±0.008	0.675±0.010	0.154±0.005	0.060±0.004
LSTM	0.662±0.010	0.826±0.008	0.680±0.009	0.152±0.005	0.057±0.004
Deep Ensemble	0.693±0.009	0.849±0.007	0.704±0.008	0.140±0.004	0.048±0.004
Previous Model	0.710±0.009	0.862±0.006	0.718±0.008	0.133±0.004	0.044±0.003
Proposed Model	0.764±0.006	0.895±0.005	0.760±0.005	0.109±0.002	0.030±0.002
Mode 4: Systematic Measurement Bias (Averaged across +10%, +20%, and +30% Calibration Drift)					
Logistic Regression	0.563±0.015	0.741±0.012	0.597±0.014	0.194±0.008	0.090±0.007
SVM	0.581±0.014	0.758±0.011	0.610±0.013	0.186±0.007	0.084±0.006
Random Forest	0.601±0.013	0.773±0.010	0.625±0.012	0.179±0.007	0.078±0.006
XGBoost	0.623±0.012	0.792±0.009	0.644±0.011	0.171±0.006	0.071±0.005
LightGBM	0.632±0.012	0.799±0.009	0.652±0.010	0.167±0.006	0.068±0.005
DNN	0.649±0.011	0.814±0.008	0.668±0.010	0.159±0.005	0.064±0.004
LSTM	0.655±0.010	0.821±0.008	0.674±0.009	0.156±0.005	0.061±0.004
Deep Ensemble	0.686±0.009	0.843±0.007	0.696±0.008	0.144±0.004	0.051±0.004
Previous Model	0.703±0.009	0.856±0.006	0.712±0.008	0.137±0.004	0.047±0.003
Proposed Model	0.752±0.007	0.886±0.005	0.750±0.006	0.115±0.003	0.033±0.002
Proposed Framework Fine-Grained Performance across Specific Severity Tiers:					
Random Missingness (10%/25%/50%): PR-AUC: 0.778/0.764/0.742; ROC-AUC: 0.902/0.894/0.881; ECE: 0.028/0.030/0.034					
Continuous Missingness (3d/7d/14d): PR-AUC: 0.772/0.755/0.738; ROC-AUC: 0.899/0.889/0.877; ECE: 0.029/0.032/0.035					
Sensor Gaussian Noise (σ=0.1/0.2/0.5): PR-AUC: 0.781/0.766/0.746; ROC-AUC: 0.904/0.896/0.884; ECE: 0.028/0.030/0.033					
Systematic Bias (+10%/+20%/+30%): PR-AUC: 0.775/0.751/0.730; ROC-AUC: 0.900/0.887/0.872; ECE: 0.029/0.032/0.037					

**Table 6:** Ablation study of the proposed framework. CIR denotes cross-source interaction representation, CCA denotes climate-constrained augmentation, and USW denotes uncertainty-guided stabilized weighting. Results are reported as mean ± 95% confidence interval over five independent runs.

Configuration	PR-AUC ↑	ROC-AUC ↑	F1-Score ↑	Brier Score ↓	ECE ↓
Base Model	0.721±0.009	0.862±0.006	0.718±0.008	0.132±0.004	0.049±0.004
Base + CIR	0.746±0.008	0.879±0.005	0.739±0.007	0.121±0.003	0.043±0.003
Base + CCA	0.741±0.008	0.875±0.005	0.735±0.007	0.124±0.003	0.045±0.003
Base + USW	0.738±0.008	0.872±0.006	0.731±0.007	0.117±0.003	0.039±0.003
Base + CIR + CCA	0.768±0.007	0.892±0.005	0.758±0.006	0.111±0.003	0.036±0.003
Base + CIR + USW	0.772±0.007	0.895±0.004	0.762±0.006	0.106±0.003	0.032±0.002
Base + CCA + USW	0.765±0.007	0.889±0.005	0.754±0.006	0.109±0.003	0.034±0.002
Full Model w/o Weight Stabilization	0.779±0.009	0.901±0.006	0.769±0.008	0.105±0.004	0.035±0.004
Full Model (CIR + CCA + USW)	0.791±0.006	0.909±0.004	0.781±0.005	0.098±0.002	0.027±0.002

**Table 7:** Sensitivity analysis of default risk prediction performance under varying spatial matching configurations on the test set. Results are reported as mean ± 95% confidence interval over five independent runs.

Spatial Matching Configuration	PR-AUC ↑	ROC-AUC ↑	F1-Score ↑	Brier Score ↓	ECE ↓
Single Nearest Station (N=1)	0.774±0.007	0.896±0.005	0.763±0.006	0.106±0.003	0.033±0.002
Multi-Station IDW (N=3,α=0.5)	0.783±0.006	0.903±0.004	0.773±0.005	0.102±0.002	0.029±0.002
Multi-Station IDW (N=3,α=1.0, Default)	0.791±0.006	0.909±0.004	0.781±0.005	0.098±0.002	0.027±0.002
Multi-Station IDW (N=3,α=1.5)	0.790±0.006	0.908±0.004	0.780±0.005	0.099±0.002	0.027±0.002
Multi-Station IDW (N=3,α=2.0)	0.788±0.006	0.907±0.004	0.778±0.005	0.100±0.002	0.028±0.002
Multi-Station IDW (N=5,α=1.0)	0.786±0.007	0.905±0.005	0.776±0.006	0.101±0.003	0.029±0.002

**Table 8:** Sensitivity analysis under varying synthetic label noise rates and augmentation ratios on the test set. Results are reported as mean ± 95% confidence interval over five independent runs.

Experimental Setting	PR-AUC ↑	ROC-AUC ↑	F1-Score ↑	Brier Score ↓	ECE ↓
Synthetic Label Flip Noise Rate (Augmentation Ratio = 30%)					
Noise Rate 0% (Default)	0.791±0.006	0.909±0.004	0.781±0.005	0.098±0.002	0.027±0.002
Noise Rate 5%	0.789±0.006	0.907±0.004	0.779±0.005	0.099±0.002	0.028±0.002
Noise Rate 10%	0.785±0.007	0.904±0.005	0.775±0.006	0.102±0.002	0.029±0.002
Noise Rate 15%	0.778±0.007	0.898±0.005	0.768±0.006	0.106±0.003	0.031±0.003
Noise Rate 20%	0.768±0.008	0.890±0.005	0.759±0.007	0.111±0.003	0.034±0.003
Noise Rate 15% (w/o USW)	0.742±0.009	0.871±0.006	0.736±0.008	0.123±0.004	0.046±0.004
Augmentation Ratio (Label Noise Rate = 0%)					
Ratio 10%	0.776±0.007	0.897±0.005	0.766±0.006	0.106±0.003	0.031±0.002
Ratio 20%	0.784±0.006	0.903±0.004	0.774±0.005	0.101±0.002	0.029±0.002
Ratio 30% (Default)	0.791±0.006	0.909±0.004	0.781±0.005	0.098±0.002	0.027±0.002
Ratio 40%	0.788±0.007	0.906±0.004	0.778±0.005	0.100±0.002	0.028±0.002
Ratio 50%	0.782±0.007	0.902±0.005	0.772±0.006	0.103±0.003	0.030±0.003

**Table 9:** Performance comparison on external validation settings: retrospective real disaster subsets, external temporal cohort (2025), and cross-regional transfer. Results are reported as mean ± 95% confidence interval over five independent runs.

Evaluation Setting	Model	PR-AUC ↑	ROC-AUC ↑	F1-Score ↑	Brier Score ↓	ECE ↓
Real Disaster: Heatwave (July 2023 Subset)	LightGBM	0.672±0.011	0.826±0.008	0.680±0.010	0.148±0.005	0.059±0.004
	LSTM	0.704±0.010	0.849±0.007	0.709±0.009	0.137±0.004	0.051±0.004
	Previous Model	0.743±0.008	0.875±0.005	0.739±0.007	0.120±0.003	0.041±0.003
	Proposed Model	0.782±0.007	0.902±0.004	0.773±0.006	0.103±0.002	0.029±0.002
Real Disaster: Flood (August 2023 Subset)	LightGBM	0.665±0.012	0.819±0.009	0.673±0.011	0.152±0.006	0.062±0.005
	LSTM	0.697±0.010	0.842±0.008	0.702±0.009	0.141±0.005	0.054±0.004
	Previous Model	0.736±0.009	0.869±0.006	0.732±0.008	0.124±0.004	0.043±0.003
	Proposed Model	0.776±0.007	0.898±0.005	0.767±0.006	0.106±0.003	0.031±0.002
Temporal External (2025 Holdout Cohort)	LightGBM	0.678±0.010	0.829±0.007	0.686±0.009	0.145±0.005	0.057±0.004
	LSTM	0.701±0.009	0.846±0.006	0.706±0.008	0.135±0.004	0.050±0.003
	Previous Model	0.731±0.008	0.866±0.005	0.728±0.007	0.126±0.003	0.044±0.003
	Proposed Model	0.774±0.007	0.897±0.004	0.766±0.006	0.107±0.003	0.030±0.002
Cross-Regional Transfer (Train: SJZ, Test: HD)	LightGBM	0.658±0.011	0.812±0.008	0.669±0.010	0.156±0.005	0.065±0.004
	LSTM	0.689±0.010	0.835±0.007	0.694±0.009	0.144±0.004	0.056±0.004
	Previous Model	0.724±0.009	0.860±0.006	0.720±0.008	0.129±0.004	0.046±0.003
	Proposed Model	0.769±0.007	0.893±0.005	0.761±0.006	0.109±0.003	0.032±0.002

**Table 10:** Global feature importance ranking and temporal observation window SHAP attributions |ϕ| for agricultural loan default risk prediction.

Feature Category	Feature Name	Overall |ϕ|	30-Day Window	60-Day Window	90-Day Window
Borrower Financial & Operational	Historical Overdue Frequency	0.412	–	–	–
	Debt-to-Income Ratio (DTI)	0.345	–	–	–
	Operational Scale (Cultivated Land Area)	0.278	–	–	–
	Irrigation Infrastructure Endowment	0.261	–	–	–
	Internal Credit Rating Tier	0.234	–	–	–
Environmental Sensing & Exposure	Consecutive High-Temperature Days ($T_{\max}>35\;{}^{\circ}C$)	0.318	0.112	0.384	0.216
	Root-Zone Soil Moisture Deficit (SMA)	0.294	0.365	0.282	0.124
	Cumulative Precipitation Anomaly (*P*)	0.252	0.098	0.291	0.312
	Field-Level NDVI Degradation Rate	0.229	0.314	0.186	0.082
	Flood Exposure Intensity Indicator (FII)	0.187	0.245	0.142	0.071

## Data Availability

The original contributions presented in this study are included in the article. Further inquiries can be directed to the corresponding author.
